# Mutations that positively affect *Bandavirus* glycoprotein function in VSV-vectored vaccines

**DOI:** 10.1128/jvi.01311-26

**Published:** 2026-09-24

**Authors:** Raegan J. Petch, Philip Hicks, Jonna B. Westover, Brian B. Gowen, Paul Bates

**Affiliations:** 1 Department of Microbiology, Perelman School of Medicine, University of Pennsylvania 6572, Philadelphia, Pennsylvania, USA; 2 Department of Animal, Dairy, and Veterinary Sciences, Utah State University 4606, Logan, Utah, USA; University of Kentucky College of Medicine, Lexington, Kentucky, USA

**Keywords:** bandavirus, severe fever with thrombocytopenia syndrome virus, SFTSV, Dabie bandavirus, Heartland bandavirus, vaccine, immunogenicity, neutralizing antibodies, passive transfer, rVSV, vesicular stomatitis virus, COPI, HRTV

## Abstract

Severe fever with thrombocytopenia syndrome virus (SFTSV) and Heartland bandavirus (HRTV) are emerging tick-borne bandaviruses. They have high case fatality rates (10%), and no FDA-approved vaccines exist for disease prevention. SFTSV and HRTV are therefore identified as priority pathogens. A recombinant vesicular stomatitis virus (rVSV) vaccine, which replaces the original VSV glycoprotein with the SFTSV glycoproteins, shows early promise for SFTSV as it induces strong immune responses that are protective against lethal challenge. However, rVSV-SFTSV is highly attenuated in cell culture, which may be due to incompatibility between the assembly sites of SFTSV (the Golgi and endoplasmic reticulum-Golgi intermediate compartment [ERGIC]) and those of VSV (the plasma membrane). In this study, we identify a noncanonical coatomer complex I (COPI)-binding motif found in the cytoplasmic tail of SFTSV glycoproteins and demonstrate that an amino acid substitution in this motif (K1071A) inhibits binding to COPI. This mutation results in increased surface expression of SFTSV glycoproteins, improved incorporation onto VSV virions, and enhanced replication of rVSV-SFTSV *in vitro*. A mutation at a homologous site (K1074A) in HRTV yields similar results, and rVSV-HRTV K1074A exhibits increased replication *in vitro* and *in vivo*. We show that vaccination with rVSV-HRTV K1074A results in improved induction of neutralizing antibody responses in immunocompetent C57BL/6 mice, and neutralizing antibodies elicited by vaccination are protective when administered to severely immunocompromised mice via passive transfer. Overall, our study identifies a mutation that improves the efficacy of the rVSV-SFTSV vaccine candidate and introduces the first vaccine candidate directly addressing HRTV infections.

**IMPORTANCE:**

Severe fever with thrombocytopenia syndrome virus (SFTSV) and Heartland bandavirus (HRTV) are emerging tick-borne viruses with high fatality rates. FDA-approved vaccines and antiviral drugs are unavailable but critically needed. We identify an important mutation in the SFTSV glycoprotein that disrupts a previously unreported COPI-binding site. The mutation improves the efficacy of the previously described recombinant vesicular stomatitis virus vaccine candidate for SFTSV (rVSV-SFTSV). We also develop an rVSV-HRTV vaccine and show potent induction of neutralizing antibodies and protection from lethal challenge. This is the first study directly addressing the lack of vaccines specifically targeting HRTV.

## INTRODUCTION

Severe fever with thrombocytopenia syndrome virus (SFTSV, recently renamed Dabie bandavirus) and Heartland bandavirus (HRTV) are emerging tick-borne viruses in the *Bunyaviracetes* class and the *Bandavirus* genus (1–3). SFTSV was first identified in China in 2009, and more than 30,000 cases have been recognized throughout East and Southeast Asia since its discovery (1, 4–16). Annual infections have increased since the virus was identified (4–6, 17), and incidences of zoonotic and human-to-human transmission have been documented with increasing frequency (18–25). However, tick bites are still the primary mode of transmission (26). Patients infected with SFTSV develop symptoms ranging from mild flu-like illness to severe disease, including high fever, thrombocytopenia, hemorrhagic manifestations, and neurological symptoms that can progress to multiorgan failure and death (1, 5, 27). The overall case fatality rate of SFTSV is approximately 8% (4), but regional outbreaks have had fatality rates as high as 47% (10). Given the continued threat of SFTSV, the high mortality, and the potential for human-to-human transmission, SFTSV was designated as a priority pathogen by the World Health Organization (WHO) and the National Institute of Allergy and Infectious Diseases (NIAID) (28, 29). Cases of SFTSV have not yet been confirmed outside of its endemic range in Southeast Asia, but HRTV, a virus that is very closely related to SFTSV, was discovered in the United States in 2009 (2). Since then, more than 60 cases of HRTV infection have been detected throughout the Eastern US (30, 31). However, HRTV is no longer a reportable disease in the US, and this case count is thought to be an underestimate of the true prevalence (30, 32, 33). Seroprevalence of the virus in wildlife is high throughout the Eastern US, and there are concerns that the virus is circulating more widely without detection (32–36). Patients infected with HRTV have similar clinical signs to those with SFTSV, and the overall case fatality rate of confirmed cases is approximately 5%–10% (31). Similar to SFTSV, HRTV has been identified as a priority pathogen by NIAID (29). Despite these designations, no vaccines or therapeutics have been approved to prevent or treat SFTSV or HRTV infections (37).

As members of the *Bunyaviracetes* class, SFTSV and HRTV have tri-segmented, single-stranded RNA genomes (1, 2). The small segment (S) is ambisense and encodes the nucleoprotein in the negative sense and a non-structural protein in the positive sense. The large (L) and medium (M) segments are negative sense and encode the RNA-dependent RNA polymerase and envelope glycoproteins (GPs), respectively. The glycoprotein is translated as a polyprotein and is proteolytically cleaved into Gn and Gc (38). Gn is predominantly responsible for recognition of the receptor, resulting in binding and attachment to cells. Following attachment, Gc mediates fusion of the viral envelope with the cell membrane and subsequent entry into cells (39–43). A definitive receptor for SFTSV has not yet been defined, but DC-SIGN, glucosylceramide, UGCG, non-muscle myosin heavy chain IIA, and CCR2 have been proposed as important entry factors (39, 40, 42, 44–46). Receptor binding and entry are less well understood for HRTV, but C-type lectins, DC-SIGN, DC-SIGNR, LSECTin, glucosylceramide, and UGCG have been shown to be involved (44, 46, 47).

Given that the glycoproteins are responsible for attachment and entry into cells, they are the primary targets of protective immune responses, and anti-Gn neutralizing antibodies are a correlate of protection in patients infected with SFTSV (48–53). Therefore, the majority of SFTSV vaccines in early stages of development utilize Gn and Gc as immunogens, and development of neutralizing antibodies in response to vaccination is the primary metric of success. T-cell responses to SFTSV proteins in the absence of neutralizing antibodies have been shown to be protective in one vaccination study, but the role of cellular immunity in controlling natural infection is not well understood (54). DNA, mRNA, protein subunit, whole inactivated virus, live attenuated virus, and virus-vectored vaccines have been developed for SFTSV, and the majority of these candidates provide immunity that is protective against lethal challenge in animal models (54–67). However, almost all of the candidates require at least two doses for complete immunity, which is a hindrance to effective vaccination in rural areas where tick exposure is most common, and vaccination is most urgently needed (68, 69). The recombinant vesicular stomatitis virus vaccine (rVSV-SFTSV) has unique advantages because it only requires one dose for complete protection and maintains a positive safety profile (63, 64). rVSV-SFTSV has also been shown to have efficacy in cross-protection against lethal HRTV challenge, which is of high importance, given that no studies directly evaluating HRTV vaccines have been published (64).

Vesicular stomatitis virus (VSV) is a rhabdovirus that has been developed as a vaccine vector due to its ability to incorporate foreign glycoproteins onto virions and induce strong, protective antibody and T-cell responses against the foreign antigen (70). An FDA-approved recombinant VSV vaccine for Zaire Ebola virus (rVSV-ZEBOV) has been used with remarkable success. A single dose of rVSV-ZEBOV results in rapid development of long-lived protective antibody and T-cell responses (71–74). Importantly, rVSV-ZEBOV was also shown to be protective if given shortly after exposure in animal studies and some cases of incidental human exposure, which is an attribute that few other vaccines have achieved (75–81). The safety of rVSV-ZEBOV is well documented, and it has now been approved for use in children (82). The incredible success of the rVSV-ZEBOV vaccine sets a precedent for the use of this platform for emerging diseases, including SFTSV and HRTV.

rVSV-SFTSV is a promising vaccine candidate, but further studies are required to optimize the virus and enhance our understanding of its therapeutic value. rVSV-SFTSV is attenuated in cell culture, and data from our lab have shown that titers of rVSV-SFTSV are approximately 90-fold lower than those of the parent VSV at 24 h post-infection (hpi) (64). Recovery of rVSV-SFTSV through plasmid-based reverse genetics systems is also challenging and is much less efficient than that of unaltered VSV or other rVSV encoding foreign glycoproteins such as ZEBOV. rVSV-SFTSV attenuation may be due to inefficient incorporation of SFTSV glycoproteins onto VSV particles due to incompatibility in viral assembly sites. VSV assembles at the cell membrane, but SFTSV glycoproteins are targeted to the endoplasmic reticulum-Golgi intermediate compartment (ERGIC) and Golgi for assembly and egress (83).

Like many other bunyaviruses, SFTSV Gn distributes to the Golgi when expressed independently of Gc, while Gc exhibits an ER distribution pattern (84–91). Signals in Gn are considered to dictate the localization of the Gn/Gc heterodimer since expression of Gn is sufficient to recruit Gc to the Golgi from the ER. Protein sorting signals responsible for these localization patterns are loosely recognized based on studies of other bunyaviruses, but mechanisms specific to SFTSV are not well understood. Golgi targeting signals have been mapped to the Gn cytosolic tail, transmembrane domain, or both the cytosolic tail and transmembrane domain of genetically related bunyaviruses, but generalizations to SFTSV are undermined by poor sequence conservation within these regions (88, 91–93). ER retention of bunyaviral Gc has been attributed to the C-terminus of the cytosolic tail (93–95), which is enriched in basic residues that fit or approximate well-established KxKxx or KKxx (where K is lysine and x is any amino acid) motifs known to bind coatomer complex I (COPI) (96–98). COPI participates in retrograde trafficking between the Golgi cisternae and from the Golgi to the ER (99). Previous studies have implicated or confirmed the role of COPI in the intracellular retention of some coronaviral spike glycoproteins (100–106), but direct investigation of the role of COPI in bunyavirus replication is generally lacking.

We hypothesize that the attenuation of rVSV-SFTSV may impair replication and immunogenicity *in vivo*. Data from our lab and others have identified four mutations (C617R, M749T, E982K, and K1071E) that arise spontaneously in SFTSV Gc upon passage of rVSV-SFTSV and may increase the replication of the rVSV-SFTSV virus in cell culture (64, 107). However, the mechanisms by which these mutations increase rVSV-SFTSV replication are not well understood, and synergism between these mutations has not yet been evaluated. While spontaneous acquisition of mutations may be a positive outcome for *in vitro* replication, it may present a challenge for clinical translation of the vaccine candidate due to concerns about instability of the viral genome. Moreover, spontaneous mutations may inadvertently disrupt epitopes important for immune control of circulating viruses. It is therefore favorable to use reverse genetics systems to genetically alter the SFTSV GP on rVSV to introduce stable mutations with known biological outcomes and predictable immunogenicity.

In this study, we evaluate the mutations C617R, M749T, E982K, and K1071A in the context of the SFTSV glycoprotein. We found that the combination of K1071A+M749T, K1071A+E982K, or K1071A+C617R+M749T results in increased titers of VSVΔG(SFTSV) pseudotypes. Evaluation of the mechanisms responsible shows increased stability, fusogenicity, and surface expression of the mutant SFTSV glycoproteins. The increased surface expression is striking and is caused solely by the K1071A substitution. As seen with other bunyaviruses (93, 94), we find that this mutation is sufficient to relocalize SFTSV glycoproteins from intracellular compartments to the cell surface. We also use immunoprecipitation assays to demonstrate that WT SFTSV Gc binds to β′-COP, and that the K1071A mutation decreases the strength of this interaction. To our knowledge, this is the first report providing direct evidence that COPI binding is important for bunyavirus protein localization. Furthermore, we establish that the K1071A mutation increases the efficiency of replication of rVSV-SFTSV and improves the incorporation of the SFTSV glycoprotein onto VSV virions. Mutations made in homologous sites in rVSV-HRTV result in a similar phenotype, where the same mutation in the cognate position of HRTV Gc (K1074A) also improves viral replication and surface glycoprotein expression in infected cells. Finally, we evaluate the immunogenicity of the rVSV-HRTV K1074A vaccine and demonstrate that it improves production of neutralizing antibody responses in C57BL/6 mice and exhibits increased *in vivo* replication in immunodeficient AG129 mice. We also show that neutralizing antibodies developed in response to rVSV-HRTV K1074A are sufficient to protect naïve AG129 mice from lethal challenge when administered via passive transfer.

## MATERIALS AND METHODS

### Plasmids

Cloning was performed using PCR products amplified by Q5 Hot Start High-Fidelity DNA Polymerase (New England Biolabs, M0494). PCR products were purified from agarose gels using a Monarch DNA Gel Extraction Kit (New England Biolabs, T1020) according to the manufacturer’s protocol and ligated using NEBuilder HiFi DNA Assembly Master Mix (New England Biolabs, E2621). All plasmids were grown in NEB 5-alpha-Competent *E. coli* (New England Biolabs, C2987) and purified using ZymoPURE Plasmid Miniprep Kit (Zymo Research, D4210) or GenElute HP Plasmid Maxiprep Kit (Sigma-Aldrich, NA0310-1KT). All plasmids were submitted for Nanopore sequencing prior to use (Eurofins Genomics).

#### Cloning of plasmids encoding full-length bunyavirus glycoproteins

Full-length DNA sequences encoding the codon-optimized coding sequences (gBlocks, Integrated DNA Technologies) of the M segments from SFTSV and HRTV were amplified by PCR and cloned into the mammalian expression plasmid pCG1 to produce pCG1-SFTSV-Gn/Gc and pCG1-HRTV Gn/Gc. Site-directed mutagenesis primers were designed to amplify the entirety of pCG1 and contain mutations encoding C617R, M749T, E982K, or K1071A substitutions for SFTSV or C621S, V753T, or K1074A substitutions for HRTV. In our original study, we determined that a mutation encoding a K1071E substitution occurred in the SFTSV glycoprotein upon passage of rVSV-SFTSV, but we decided to evaluate a K1071A substitution instead based on other reports of this substitution in similar positions of Rift Valley fever virus (RVFV), Uukuniemi virus (UUKV), and SARS-CoV-2, among others (93, 94, 100). Similarly, we chose to evaluate a C621S substitution in HRTV as opposed to the C621R substitution based on reports that a C621S substitution arises upon passage of HRTV in mice (108). The single PCR products for each plasmid were ligated by NEBuilder HiFi DNA assembly. Following the production of plasmids with each mutation alone, we used the same mutagenesis primers to produce plasmids with multiple mutations. In total, we produced pCG1-SFTSV Gn/Gc WT, C617R, M749T, E982K, K1071A, C617R+M749T, K1071A+C617R, K1071A+M749T, K1071A+E982K, and K1071A+C617R+M749T. We also produced pCG1-HRTV Gn/Gc WT, K1074A, and C621S+V753T+K1074A.

#### Cloning of pVSV-SFTSV launch plasmids

The pVSV eGFP RABV-G plasmid (Addgene, #31833) was used as the template for creating vectors expressing the VSV antigenome with SFTSV or HRTV glycoproteins in place of the VSV-G glycoprotein. Primers were designed to amplify the pVSV vector backbone with overlaps matching the 5′ and 3′ ends of SFTSV or HRTV glycoproteins. The SFTSV or HRTV glycoprotein (WT or with the indicated substitutions) was amplified from the pCG1 plasmids described above using primers designed to overlap with the pVSV vector backbone. NEBuilder HiFi DNA Assembly was performed to ligate the glycoprotein inserts to the pVSV vector.

#### Cloning of VSV helper plasmids for VSV launch

The pCG1 expression vector was modified to remove two T7 promoters, which were hypothesized to compete for T7-driven polymerase expression of the pVSV vector plasmid, to produce the expression plasmid pCG2. Full-length, codon-optimized sequences of VSV-N, -P, and -L were synthesized (gBlocks, Integrated DNA Technologies) based on previous reports (109) and cloned into pCG2 expression vector downstream of the rabbit beta-globin intron. T7opt in the pCAGGS vector was a gift from Benhur Lee (Addgene, #65974) (110).

#### Cloning of fluorescent SFTSV glycoprotein chimeras

Sequences encoding mCherry (amino acids 2–236) and mNeonGreen (amino acids 2–236) were amplified by PCR. PCR using pCG1-SFTSV-Gn/Gc WT or K1071A template DNA was performed to delete sequences encoding the ectodomain of Gc (amino acids 567–1,029). NEBuilder HiFi DNA Assembly was performed using reactions containing two products to generate the cloning intermediate plasmids pCG1-SFTSV-Gn/Gc^ΔEcto/mCherry^ and pCG1-SFTSV-Gn/Gc^ΔEcto/mNeonGreen^. To generate pCG1-SFTSV-Gc^ΔEcto/mCherry^ and pCG1-SFTSV-Gc^ΔEcto/mNeonGreen^, PCR was performed on pCG1-SFTSV-Gn/Gc^ΔEcto/mNeonGreen^ and pCG1-SFTSV-Gn/Gc^ΔEcto/mCherry^ to generate a product in which the sequence encoding the entirety of Gn (amino acids 1–535) was deleted. These products were circularized using NEBuilder HiFi DNA Assembly. These plasmids are hereby referred to as mNeonGreen-RxKxx, mNeonGreen-RxAxx, mCherry-RxKxx, and mCherry-RxAxx to reflect plasmids in which the ectodomain of Gc was replaced by a fluorescent reporter appended to either the WT transmembrane domain and cytoplasmic tail (-RxKxx) or the transmembrane domain and cytoplasmic tail with a lysine-to-alanine mutation in the −3 position (-RxAxx).

### Cells

ATCC-verified and mycoplasma-free HEK293T (293T), Vero E6, A549, and HeLa cells were maintained in DMEM (Corning, MT10-013-CV) with 10% cosmic calf serum (CCS) (HyClone, #SH30087.03). Cells were passaged every 2–3 days using Trypsin-EDTA (0.05%) (Invitrogen, 25300054). Where indicated, cells were seeded in plates coated with 0.025 mg/mL rat tail collagen (Corning, 354236) in 0.02 M acetic acid.

To launch rVSV-SFTSV, we developed HEK293T cells that stably expressed CCR2, which is known to be an important entry factor for SFTSV (42). A plasmid encoding human CCR2 transcript variant B was purchased from GenScript (OHu23522) and was transfected into HEK293T cells using Lipofectamine 3000 according to the manufacturer’s guidelines. One day after transfection, the cells were passaged into media containing 500 μg/mL Geneticin (Gibco, 10131-035). Surviving colonies were passaged and expanded in media with Geneticin.

### Production of VSVΔG(SFTSV) mutant pseudotypes

VSVΔG(SFTSV) pseudotypes were produced as previously described (111). Briefly, 293T cells were seeded in collagen-coated 10-cm plates in the afternoon to achieve a density of 70% confluency the following morning. Cells were transfected with 25 μg of purified DNA from plasmids encoding the SFTSV mutant glycoproteins using calcium phosphate according to the manufacturer’s protocol (Takara Bio, 631312). Approximately 24 h post-transfection (hpt), transfected cells were infected with VSVΔG-RFP virus pseudotyped with VSV-G at a multiplicity of infection (MOI) of ~1–3 at 37°C. Approximately 24 hpi, supernatant was harvested from infected cells and clarified twice by centrifugation at 1,250 × *g* for 10 min at 4°C. Purified supernatant was aliquoted and stored at −80°C.

To titer the pseudotypes, Vero E6 cells were seeded in the afternoon in 100 μL at a density of 2.5 × 10^4^ cells/well into a collagen-coated 96-well plate. The next morning, VSVΔG-RFP(SFTSV) mutant pseudotypes were serially diluted twofold in DMEM + 10% CCS. The dilution medium also contained 600 ng/mL anti-VSV-G monoclonal antibody 1E9F9, which was used to neutralize any carryover pseudovirus containing VSV-G. The virus/antibody mixtures were incubated at 37°C for 1 h before being transferred onto Vero E6 cells. After 30 h of infection, cells were gently washed with phosphate-buffered saline (PBS) and fixed with 4% paraformaldehyde. Transduced cells were detected by RFP fluorescence using an S6 FluoroSpot Analyzer (CTL, Shaker Heights, OH). Individual spots were counted using Basic Count settings with spot sizes. Average foci counts from uninfected wells were subtracted from all wells on the same plate to correct for background. Infectious titers (focus-forming units, FFU/mL) were calculated per sample by averaging values collected from all wells within the linear range on the dilution curve.

### SFTSV mutant glycoprotein surface expression flow cytometry

A549 cells were seeded into 6-well plates in the afternoon to achieve a density of 80% confluency the following morning. Cells were transfected with 2 μg of purified SFTSV mutant glycoprotein DNA using Lipofectamine 3000 according to the manufacturer’s protocol (Invitrogen, L3000-015). Cells were incubated in Opti-MEM (Gibco, 31985070) following transfection and supplemented with pre-warmed DMEM + 10% CCS for 3 hpt. Transfected cells were lifted off the plate 24 hpt using PBS with 10 mM EDTA. Cells were pelleted at 400 × *g* for 5 min at 4°C and resuspended in FACS buffer (PBS with 2% BSA and 0.1% sodium azide) containing LIVE/DEAD Fixable Violet Dead Cell Stain (Invitrogen, L34964) at a 1:40 dilution and serum from mice immunized with an mRNA LNP encoding the unaltered, full-length SFTSV Gn/Gc (112) at a dilution of 1:1,000. Cells were incubated in this LIVE/DEAD and primary antibody mixture in the dark for 1 h at room temperature to label proteins on the cell surface. Following incubation, cells were pelleted and washed three times with FACS buffer, then fixed with 4% paraformaldehyde for 20 min. Cells were briefly washed twice with FACS buffer, and then they were permeabilized with FACS buffer containing 0.1% Triton X-100 for 30 min. To label intracellular proteins, cells were incubated for an additional hour with a mixture of primary rabbit polyclonal antibodies against SFTSV Gn (ProSci, #6647) and SFTSV Gc (ProSci, #6653), each at a 1:2,000 dilution. Cells were again washed three times with FACS buffer, and then they were stained with 1:1,000 dilutions of donkey anti-mouse IgG Alexa Fluor 488 (Invitrogen, A21202) and donkey anti-rabbit IgG Alexa Fluor 594 (Invitrogen, A21207) for 1 h at room temperature. Cells were washed three final times with FACS buffer and analyzed on a BD LSRII flow cytometer with a high-throughput system using FACSDIVA software (BD Biosciences). Data analysis was performed using FlowJo software (FlowJo LLC). Gating was performed to select live single cells based on low violet fluorescent reactive dye staining. Stained mock-transfected cells were used to gate for both surface-positive and permeabilized-positive signals. The mean fluorescence intensity (MFI) of surface-positive (AF488) and permeabilized-positive (AF594) antigen was calculated, and the surface-positive MFI was compared to the MFI of total antigen (surface-positive + permeabilized-positive) to quantify the percentage of SFTSV antigens that were surface expressed.

### SFTSV mutant glycoprotein stability

293T cells were seeded into collagen-coated 6-well plates in the afternoon to achieve a density of 80% confluency the following morning. Cells were transfected in triplicate with 2 μg of purified SFTSV mutant glycoprotein DNA using Lipofectamine 3000 according to the manufacturer’s protocol (Invitrogen, L3000-015). Cells were incubated in Opti-MEM (Gibco, 31985070) following transfection and supplemented with pre-warmed DMEM + 10% CCS for 3 hpt. Forty-eight hpt, two wells of cells transfected with each glycoprotein were treated with cycloheximide (Sigma-Aldrich, C7698-1G) at a final concentration of 50 mg/mL according to established protocols (113). The third well of each condition was treated with DMSO as a vehicle control.

Six hours post-treatment, one well of cycloheximide-treated cells and the DMSO-treated cells were harvested for each SFTSV mutant. The cells were washed gently with ice-cold PBS and removed from the plate. They were pelleted at 400 × *g* for 5 min at 4°C and resuspended in 200 μL Triton X-100 cell lysis buffer with cOmplete Protease Inhibitor Cocktail (Millipore Sigma, 11836170001). Cells were incubated at 4°C for lysis, and then debris was pelleted by centrifuging at 15,000 rpm for 10 min at 4°C. The supernatant was transferred to a clean tube and stored at −20°C prior to analysis via Western blot. The above protocol was repeated for the remaining well at 10 h post-treatment.

Protein samples were quantified via NanoDrop and normalized to the same concentration prior to Western blot. Laemmli sample buffer was added, and the proteins were denatured at 95°C for 5 min. Samples were run on a 4%–15% Criterion Tris-HCl Protein Gel (Bio-Rad Laboratories, 3450028). Protein was transferred to an Amersham Hybond P 0.45 PVDF membrane (Cytiva, 10600029) and blocked in 5% milk in TBS buffer with 0.1% Tween-20 (TBS-T) for 1 h. Proteins were probed with rabbit polyclonal anti-SFTSV Gc antibody at a 1:5,000 dilution (Pro Sci, #6651) and a mouse monoclonal anti-hGAPDH antibody at a 1:500 dilution (Developmental Studies Hybridoma Bank, DSHB-GAPDH-2G7) overnight at 4°C. The membranes were washed three times with TBS-T and then incubated with HRP-conjugated secondary antibodies (Invitrogen 31430—1:100,000 and A27036—1:30,000) for 20 min at room temperature. The membranes were again washed three times with TBS-T and then developed using SuperSignal West Femto Maximum Sensitivity Substrate (Thermo Scientific, 34095) and read on a GE Healthcare Amersham 600 imager. Band intensity was analyzed via FIJI (ImageJ).

### SFTSV mutant glycoprotein fusogenicity

HeLa cells were seeded into collagen-coated 6-well plates in the afternoon to achieve a density of 80% confluency the following morning. Cells were transfected with 2 μg of purified SFTSV mutant glycoprotein DNA using Lipofectamine 3000 according to the manufacturer’s protocol (Invitrogen, L3000-015). Cells were incubated in Opti-MEM (Gibco, 31985070) following transfection and supplemented with pre-warmed DMEM + 10% CCS for 3 hpt. Twenty-four hpt, cells were removed from the plates using Trypsin-EDTA (0.05%) (Invitrogen, 25300054) and seeded in new collagen-coated 6-well plates to achieve a monolayer the following day. The next day (48 hpt), the medium was removed, and cells were washed twice with PBS to remove all medium. They were treated with PBS buffered with 50 mM MES monohydrate (Thermo Scientific, A16104.22) adjusted to pH 5.5 for 5 min. Following the low pH incubation, the acidic buffer was removed, the cells were washed twice more with PBS, and then 2 mL of pre-warmed DMEM + 10% CCS was added to the cells. They were incubated for 4 h, and then brightfield images of five high-powered fields per condition were taken. Syncytia per high-powered field and nuclei per syncytia were counted for each condition.

### Fluorescence microscopy

A549 cells were seeded onto collagen-coated 96-well black plates with #1.5 high-performance cover glass (0.17 ± 0.005 mM) bottoms in the afternoon. The following morning, cells at a confluency of ~90% were transfected with purified plasmid DNA using Lipofectamine 3000 (Invitrogen, L3000-015) according to the manufacturer’s protocol. The medium was replaced on cells 6 hpt. Cells were gently washed with PBS 24 hpt and were subsequently fixed with 4% paraformaldehyde. In the indicated experiments, cells were treated with 33 μM nocodazole (Thermo Scientific, AC358240100) for 3 h, then washed and fixed 24 hpt.

To stain cells for immunofluorescence microscopy, fixed cells were first blocked for 1 h in blocking buffer (PBS with 0.1% sodium azide and 10% heat-inactivated donkey serum [Sigma, D9663-10 mL]) at room temperature. In assays specifically analyzing surface antigen, unpermeabilized cells were covered in 50 μL SFTSV-vaccinated mouse serum diluted 1:5,000 in staining buffer (PBS with 0.1% sodium azide and 5% donkey serum) and incubated for 1 h at room temperature. Cells were washed three times with staining buffer before being fixed again with 4% paraformaldehyde and permeabilized with blocking buffer containing 0.1% Triton X-100 for 30 min at room temperature. Intracellular antigen was labeled by staining with anti-SFTSV Gc rabbit polyclonal antibody (Pro Sci, #6653) diluted 1:1,000 in staining buffer for 1 h at room temperature. In the indicated experiments, β′-COP was labeled with a polyclonal rabbit antibody at a dilution of 1:500 (Invitrogen, PA5-96557). Cells were washed three times and then stained with secondary antibodies (Thermo Scientific, A-21125 and A21206) at a 1:1,000 dilution for 1 h at room temperature. All cells were stained using 10 μM Hoechst 33258 (Enzo Life Sciences, ENZ-52402) diluted in PBS for 30 min. Images were captured on a Nikon Eclipse Ti2-E inverted microscope (Nikon, Minato City, Tokyo, Japan) using an ORCA-flash4.0 V3 digital CMOS camera (Hamamatsu Photonics K.K., Hamamatsu City, Japan, #C13440). Imaging at 100x magnification was performed with immersion oil type F (Nikon, MXA22168). Laser intensities and exposure times were held constant across a single experiment to permit downstream quantitative analysis. Images were deconvolved using NIS-Elements software (Nikon, Minato City, Tokyo, Japan) with automatic settings.

Images were analyzed and prepared for publication using FIJI (ImageJ) software (114). Brightness/contrast was adjusted and held constant across a single experiment. Colocalization was quantified by fractional overlap on representative equatorial slices of z-stack images (115). Briefly, a region of interest was drawn around each individual cell. To perform background subtraction, pixel intensity was measured in the nuclear region of each cell, and the median pixel intensity was subtracted globally from each ROI using the Subtract command. This process was performed iteratively until the median pixel intensity for the nuclear region was 0. Channels were split, and an automatic local threshold was applied to each with a Bernsen radius of 5. Channel images were then converted to binary masks. Binary images of two channels were multiplied together using the Image Calculator to create a binary image of overlapping pixels. Signal area of all relevant binary images was measured using the Analyze Particles command and size inclusion criteria of 5 to infinity pixels. Fractional overlap coefficients were calculated by dividing total overlapping pixel area by the pixel area of the channel of interest.

### mCherry flow cytometry

The surface expression of mCherry-RxKxx, mCherry-RxAxx, or cytosolic mCherry was evaluated using flow cytometry, similar to the methods described above. Briefly, A549 cells were seeded in 6-well plates in the afternoon to achieve a density of 80% confluence the following morning. Cells were transfected with 1 μg of purified mCherry-RxKxx, mCherry-RxAxx, or cytosolic mCherry plasmid DNA using Lipofectamine 3000 as described above. Transfected cells were harvested 24 hpt using PBS with 10 mM EDTA. Cells were pelleted at 400 × *g* for 5 min at 4°C and resuspended in FACS buffer (PBS with 2% BSA and 0.1% sodium azide) containing LIVE/DEAD Fixable Violet Dead Cell Stain (Invitrogen, L34964) at 1:20 dilution for 30 min. Following incubation, cells were pelleted and washed three times with FACS buffer, then fixed with 4% paraformaldehyde for 20 min. Cells were briefly washed twice with FACS buffer and then were resuspended in FACS buffer containing a mouse monoclonal antibody against mCherry (BioLegend, 677702) and donkey anti-mouse IgG Alexa Fluor 488 (Invitrogen, A21202) for 2 h. Cells were washed three final times with FACS buffer and analyzed on a BD LSRII flow cytometer with a high-throughput system using FACSDIVA software (BD Biosciences). Data analysis was performed using FlowJo software (FlowJo LLC). Gating was performed to select live single cells based on low violet fluorescent reactive dye staining. The baseline autofluorescence of mock-transfected cells was used to subtract background fluorescence from all conditions. The MFI of surface-positive (AF488) and intracellular (mCherry) antigen was calculated, and the surface-positive MFI was compared to the MFI of total antigen (surface-positive + intracellular-positive) to quantify the percentage of SFTSV antigens that were surface expressed.

### β′-COP immunoprecipitation

293T cells were seeded in collagen-coated 10-cm plates in the afternoon to achieve a density of 80% confluence the following morning. Cells were transfected with 5 μg of purified SFTSV mutant glycoprotein DNA and 5 μg of purified β′-COP DNA with a C-terminal FLAG tag (GenScript, OHu04585D) using Lipofectamine 3000 according to the manufacturer’s protocol (Invitrogen, L3000-015). Cells were incubated in Opti-MEM (Gibco, 31985070) following transfection and supplemented with pre-warmed DMEM + 10% CCS for 3 hpt. Forty-eight hpt, the cells were washed gently with ice-cold PBS and removed from the plate. They were pelleted at 400 × *g* for 5 min at 4°C and resuspended in Triton X-100 cell lysis buffer with cOmplete Protease Inhibitor Cocktail (Millipore Sigma, 11697498001). Cells were incubated at 4°C for lysis, and then debris was pelleted by centrifuging at 15,000 rpm for 10 min at 4°C. The supernatant was transferred to a clean tube and stored at −20°C prior to analysis via Western blot and immunoprecipitation.

For immunoprecipitation, 1 mL of lysate containing the SFTSV mutant glycoprotein and the FLAG-tagged β′-COP plasmid was transferred to a separate tube containing 100 μL of Pierce Anti-DYKDDDDK Magnetic Agarose (Thermo Scientific, A36797). The tubes containing the lysate and the magnetic beads were incubated on an inverter overnight at 4°C. The rest of the lysate was used for the “input” Western blot to ensure that equal amounts of protein were expressed for each condition. Following overnight incubation, beads were isolated using a magnetic rack and washed three times with Triton X-100 lysis buffer containing cOmplete Protease Inhibitor Cocktail (Millipore Sigma, 11697498001). Seventy microliters of Laemmli sample buffer was added to the beads for each condition, and proteins were denatured at 95°C for 5 min to elute the protein from the beads.

Immunoprecipitation samples were run on a 4%–15% Criterion Tris-HCl Protein Gel (Bio-Rad Laboratories, 3450028). Protein was transferred to an Amersham Hybond P 0.45 PVDF membrane (Cytiva, 10600029) and blocked in 5% milk in TBS buffer with 0.1% Tween-20 (TBS-T) for 1 h. Proteins were probed with rabbit polyclonal anti-SFTSV Gc antibody at a 1:5,000 dilution (Pro Sci, #6651) overnight at 4°C. The membranes were washed three times with TBS-T and then incubated with a highly cross-adsorbed HRP-conjugated anti-rabbit secondary antibody (Invitrogen A16110) at a dilution of 1:20,000 for 20 min at room temperature. The membranes were again washed three times with TBS-T and then developed using SuperSignal West Femto Maximum Sensitivity Substrate (Thermo Scientific, 34095) and read on a GE Healthcare Amersham 600 imager. Input samples were run in the same way but were stripped with Restore PLUS Western Blot Stripping Buffer (Thermo Scientific, 46430) after the SFTSV probe. They were reprobed with a rabbit β′-COP polyclonal antibody (Invitrogen, PA5-96557) at a 1:6,000 dilution and then washed and incubated with the highly cross-adsorbed HRP-conjugated antibody as described above.

### rVSV-SFTSV and rVSV-HRTV launch

Recombinant VSV-SFTSV and VSV-HRTV viruses with an additional open reading frame encoding eGFP in the first position were launched as described previously (109). Briefly, 293T cells that were designed to stably express CCR2 were seeded in collagen-coated 6-well plates in the afternoon to reach a density of 60% confluence the following morning. Cells were transfected with purified DNA from plasmids encoding the VSV genome, VSV-N, VSV-P, VSV-L, and T7 polymerase at a 1:3:1:1:2 molar ratio with a total of 2.7 μg DNA per well. Transfections were done with Lipofectamine 3000 according to the manufacturer’s protocol (Invitrogen, L3000-015). Cells were incubated in Opti-MEM (Gibco, 31985070) following transfection and supplemented with pre-warmed DMEM with 10% CCS for 3 h post-transfection. Cells were watched closely over a period of 5–7 days, and the medium was replaced with pre-warmed DMEM + 10% CCS whenever the medium appeared spent. Cells were observed for eGFP expression and cytopathic effect, including cell rounding and death. Supernatant was harvested from cells once most cells were infected and clarified via centrifugation at 1250 × *g* for 10 min at 4°C; the clarified supernatant was then passaged onto Vero E6 cells to produce passage 1 of each virus.

Large stocks of each rVSV-SFTSV and rVSV-HRTV virus were grown in Vero E6 cells by infecting a confluent 15-cm plate at an MOI of 0.0001. Virus-containing supernatants were collected 48–72 hpi and were clarified by centrifuging twice at 1,250 × *g* for 5 min at 4°C. Supernatants were then passed through a syringe filter with 0.22 μm pore size (Corning, 431219) to remove cellular debris. The virus was then frozen at −80°C until used for ultracentrifugation. Virus was concentrated by ultracentrifugation of virus-containing media through a sucrose cushion at 26,000 rpm for 2 h at 4°C using SW-32 tubes in a Beckman Coulter Optima XPN-80 ultracentrifuge (Beckman Coulter). After removal of the sucrose and media, the pelleted virus was placed on ice with 1 mL sterile TBS per 15-cm plate overnight. The next day, virus pellets were gently resuspended, aliquoted, and frozen at −80°C. Viral titer was determined by plaque assays on Vero E6 cells with a 1.25% Avicel RC-591 NF (DuPont, #RC591-NFBA500) overlay and then stained with 1% crystal violet. To assess the genetic stability of the glycoproteins, all rVSV-SFTSV and rVSV-HRTV viruses were passaged on Vero E6 cells for a minimum of five passages. Viral RNA was collected and extracted, and cDNA from each sample was amplified via PCR and sequenced via Nanopore Sequencing Technology (Eurofins Genomics).

Replication kinetics were evaluated via a standard replication time-course experiment. Briefly, Vero E6 cells were seeded in 10-cm plates in the afternoon to achieve a monolayer the following morning. The next day, the cells were infected at an MOI of 0.001. Supernatant samples were collected at 12, 24, 48, and 72 h post-infection, and the media removed was replaced with an equivalent volume of pre-warmed DMEM with 10% CCS. Viral titers of each virus at each timepoint were quantified via plaque assays on Vero E6 cells. Plaques produced by each virus were measured 96 hpi with a standard ruler and rounded to the nearest quarter of a millimeter.

### Glycoprotein surface expression in rVSV-SFTSV and rVSV-HRTV-infected cells

Similar to the methods described above, the surface expression of glycoproteins in rVSV-SFTSV and rVSV-HRTV-infected cells was evaluated by flow cytometry. A549 cells were seeded in collagen-coated plates in the afternoon to achieve a monolayer the following morning. Cells were infected with the rVSV-SFTSV and rVSV-HRTV mutants at an MOI of 2. rVSV-SFTSV M749T and rVSV-HRTV WT (p2) could not be concentrated to a titer that would permit infection at the same MOI, and the replication kinetics of these viruses prohibited analysis at the same timepoint as the other viruses. For this reason, these samples were excluded from these analyses. Twenty-four hours after infection, samples were harvested using PBS with 10 mM EDTA and prepared for flow cytometry. As described above, cells were pelleted at 400 × *g* for 5 min at 4°C and resuspended in FACS buffer containing LIVE/DEAD Fixable Violet Dead Cell Stain (Invitrogen, L34964) at 1:40 dilution and serum from mice vaccinated against SFTSV glycoproteins or HRTV glycoproteins. Cells were incubated in this LIVE/DEAD and primary antibody mixture in the dark for 1 h at room temperature to label proteins on the cell surface. Following incubation, cells were pelleted and washed three times with FACS buffer, then fixed with 4% paraformaldehyde for 20 min. Cells were briefly washed twice with FACS buffer, and then they were stained with a 1:1,000 dilution of donkey anti-mouse IgG Alexa Fluor 594 (Invitrogen, A21203) for 1 h at room temperature. Cells were washed three final times with FACS buffer and analyzed on a BD LSRII flow cytometer with a high-throughput system using FACSDIVA software (BD Biosciences). Data analysis and flow cytometry plot construction were performed using FlowJo software (FlowJo LLC). Gating was performed to select live single cells based on low violet fluorescent reactive dye staining and infected cells via positive GFP staining. Stained mock-infected cells were used to gate for both infected (GFP) and surface antigen-positive (Alexa Fluor 594) signal. The MFI of surface-positive antigen in all infected cells was calculated to quantify the total surface expression of SFTSV or HRTV glycoproteins in infected cells.

### Quantification of glycoprotein incorporation into virions

Western blot analysis was used to quantify SFTSV and HRTV glycoprotein incorporation into purified VSV virions. Triton X-100 lysis buffer with cOmplete Protease Inhibitor Cocktail (Millipore Sigma, 11697498001) was added directly to purified virions. Laemmli sample buffer was added, and the proteins were denatured at 95°C for 5 min. Samples were run on a 4%–15% Criterion Tris-HCl Protein Gel (Bio-Rad Laboratories, 3450028). Protein was transferred to an Amersham Hybond P 0.45 PVDF membrane (Cytiva, 10600029) and blocked in 5% milk in TBS buffer with 0.1% Tween-20 (TBS-T) for 1 h. Proteins were probed with rabbit polyclonal anti-SFTSV Gc antibody at a 1:5,000 dilution (Pro Sci, #6651), rabbit polyclonal anti-SFTSV Gn antibody at a 1:3,000 dilution (Pro Sci, #6647), and a mouse monoclonal anti-VSV-M antibody at a 1:5,000 dilution (Kerafast, 23H12). The SFTSV Gn and Gc antibodies were also used to assess incorporation of HRTV glycoproteins into VSV virions because they demonstrated cross-reactivity against HRTV glycoproteins. Membranes were stripped with Restore PLUS Western Blot Stripping Buffer (Thermo Scientific, 46430) between each antibody. Proteins were detected with HRP-conjugated secondary antibodies (Invitrogen 31430—1:100,000 and A27036—1:30,000) for 20 min at room temperature, and the membranes were developed using SuperSignal West Femto Maximum Sensitivity Substrate (Thermo Scientific, 34095) and read on a GE Healthcare Amersham 600 imager. Band intensity was analyzed via FIJI (ImageJ).

### rVSV-HRTV vaccination studies

To evaluate neutralizing antibody responses to rVSV-HRTV in immunocompetent mice, we vaccinated five male and five female 8-week-old C57BL/6 mice (The Jackson Laboratory, #000664). The mice were anesthetized with isoflurane and vaccinated intraperitoneally (IP) with 10^6^ PFU of rVSV-HRTV WT (p3), K1074A, or C621S+V753T+K1074A at day 0 and again at day 28. The viruses were diluted in sterile PBS immediately prior to injection, and the total injection volume was 150 μL. We collected blood from mice anesthetized with isoflurane via the submandibular route, and the blood was processed immediately following collection. Briefly, serum was isolated via centrifugation at 8,000 rpm for 30 min at 4°C in an Eppendorf 5424R centrifuge (Eppendorf, Enfield, CT). EDTA was added to each sample for a final concentration of approximately 5 mM. Serum was heat inactivated by incubating at 53°C for 30 min, then centrifuged at 15,000 rpm for 20 min. Serum samples were stored at −20°C.

To evaluate protection from lethal challenge, 8-week-old AG129 mice (deficient in interferon α/β and γ receptors) were obtained from breeding colonies at Utah State University. Six male and six female mice per group were IP vaccinated with 10^2^ PFU of each rVSV-HRTV vaccine in a 100 μL inoculum. Again, the vaccines were diluted in sterile PBS immediately prior to injection. An additional control group of four male and four female mice was injected with 100 μL PBS. Mice were weighed and closely evaluated for signs of illness each day following vaccination, and approximately 50 μL of blood was collected from a subset of mice in each group 2 days post-vaccination to quantify rVSV replication via qPCR. Twenty-one days post-vaccination, 200 μL of blood was collected for neutralizing antibody analysis, and serum was isolated as indicated above. Twenty-three days post-vaccination, all mice were challenged with 10^2^ CCID_50_ (median cell culture infectious dose) of mouse-adapted HRTV delivered subcutaneously. The mouse-adapted HRTV (MA-HRTV) strain employed was derived from the MO-4 strain obtained from Dr. Robert Tesh (WRCEVA) (108). The MA-HRTV stock (5.0 × 10^6^ CCID_50_/mL; one passage in Vero E6 cells, five passages of clarified liver homogenate in AG129 mice) used was prepared from a final passage in Vero E6 cells. The virus stock was diluted in sterile MEM and inoculated via injections of 200 μL. An additional group of two male and two female mice was sham infected with 200 μL MEM for use as a control. Following challenge, mice were weighed and evaluated daily for signs of infection.

We also evaluated the protective efficacy of passive serum transfer in AG129 mice. We pooled immune serum from the day 70 bleed of C57BL/6 mice vaccinated with rVSV-HRTV WT (p3) and rVSV-HRTV K1074A and evaluated the neutralizing titer of the pool via FRNT_50_. The approximate neutralizing antibody titer of the serum from rVSV-HRTV WT (p3) mice was 25, and the neutralizing titer from the mice vaccinated with rVSV-HRTV K1074A was approximately 253. Mice were injected subcutaneously with 10^2^ CCID_50_ mouse-adapted HRTV on day 0 and then were administered serum via IP injection on day 1. Four male and four female mice were included in each group. Mice were administered 120 μL of immune serum or 120 μL of serum diluted 50% with sterile PBS, with one exception. Mice in the low-dose rVSV-HRTV K1074A group received 110 μL of serum due to constraints on the volume of serum collected. Two additional groups were included as controls, and they were administered either 120 μL of PBS or 120 μL of non-immune serum from mice who had not been immunized against HRTV. Mice were weighed and evaluated for signs of infection for 21 days post-challenge.

All mice were given approximately 1 week to acclimate to their cages and environment prior to initiation of experiments, and mice were maintained according to the Guide for Care and Use of Laboratory Animals. Vaccination experiments in C57BL/6 mice were performed under animal biosafety level 2 (ABSL-2) facilities at the University of Pennsylvania. Vaccination experiments in AG129 mice that included HRTV challenge were performed in ABSL-3 facilities at Utah State University.

### rVSV-HRTV replication RT-qPCR

To quantify replication of rVSV-HRTV WT (p3), K1074A, and C621S+V753T+K1074A, we evaluated viremia in vaccinated mice via quantitative PCR (qPCR). Fifty microliters of blood was collected from a subset of mice 2 days post-vaccination, and 300 μL of TRIzol Reagent was added to each blood sample (Invitrogen, 15596026). RNA was extracted from each sample using the manufacturer’s protocol. cDNA was prepared from each RNA sample using the SuperScript III First-Strand Synthesis System according to the manufacturer’s protocol (Invitrogen, 18080-051). The final volume of cDNA was diluted 50% with sterile nuclease-free water and used for qPCR. For each reaction, we used 4.6 μL of PowerUp SYBR Green Master Mix (Applied Biosystems, A25742), 0.2 μL of each primer (10 μM), and 5 μL of diluted cDNA. Each cDNA sample was evaluated in triplicate using primers against VSV-N (Forward: 5′-GGAATACCCGGCAGATTACTT-3′; Reverse: 5′- GCCTTGGTAGACATATCCTCTTAG-3′) and mouse hypoxanthine-guanine phosphoribosyltransferase (mHPRT) (Forward: 5′-GTTGGATACAGGCCAGACTTTGTTG-3′; Reverse: 5′-GAGGGTAGGCTGGCCTATTGGCT-3′). The qPCR assay was run in a 384-well plate on a QuantStudio 6 Flex Real-Time PCR System (Applied Biosystems). The following cycling conditions were used: hold stage: 50°C for 2 min, 95°C for 10 min; PCR stage: 40 cycles of 95°C for 15 s, 60°C for 30 s, 72°C for 30 s; and melt curve stage: 95°C for 15 s, 60°C for 1 min, 95°C for 15 s. Each assay was run using a VSV-N dilution series for positive controls and water for negative controls. The fold change of VSV-N (normalized to the mHPRT housekeeping gene) in vaccinated mice was compared to the sham control group of mice using the 2^−ΔΔCt^ method (116).

### Pseudovirus neutralization assays

Neutralizing antibodies produced in response to vaccination were evaluated via focus reduction neutralization tests (FRNTs). VSV pseudotypes with WT HRTV glycoproteins [VSVΔG-RFP(HRTV)] were produced as described above. Vero E6 cells were seeded in 100 μL at 2 × 10^4^ cells/well in a 96-well collagen-coated plate. The next day, twofold serially diluted serum samples were mixed with VSVΔG-RFP(HRTV) pseudotype virus (100–200 focus-forming units/well) and incubated for 1 h at 37°C. Also included in this mixture, to neutralize any potential VSV-G carryover virus, was 1E9F9, a mouse anti-VSV Indiana G, at a concentration of 600 ng/mL. The antibody-virus mixture was then used to replace the media on Vero E6 cells. Then, 30 hpi, the cells were washed with PBS and fixed with 4% paraformaldehyde before visualization on an S6 FluoroSpot Analyzer (CTL, Shaker Heights, OH, USA). Individual infected foci were enumerated, and the values were compared to control wells without serum. The FRNT 50% (FRNT_50_) was measured as the greatest serum dilution at which focus count was reduced by at least 50% relative to control cells that were infected with pseudotype virus in the absence of mouse serum. FRNT_50_ titers for each sample were measured in two to three technical replicates performed on separate days.

## RESULTS

### Impact of SFTSV glycoprotein mutations on glycoprotein function

**Fig 1:**
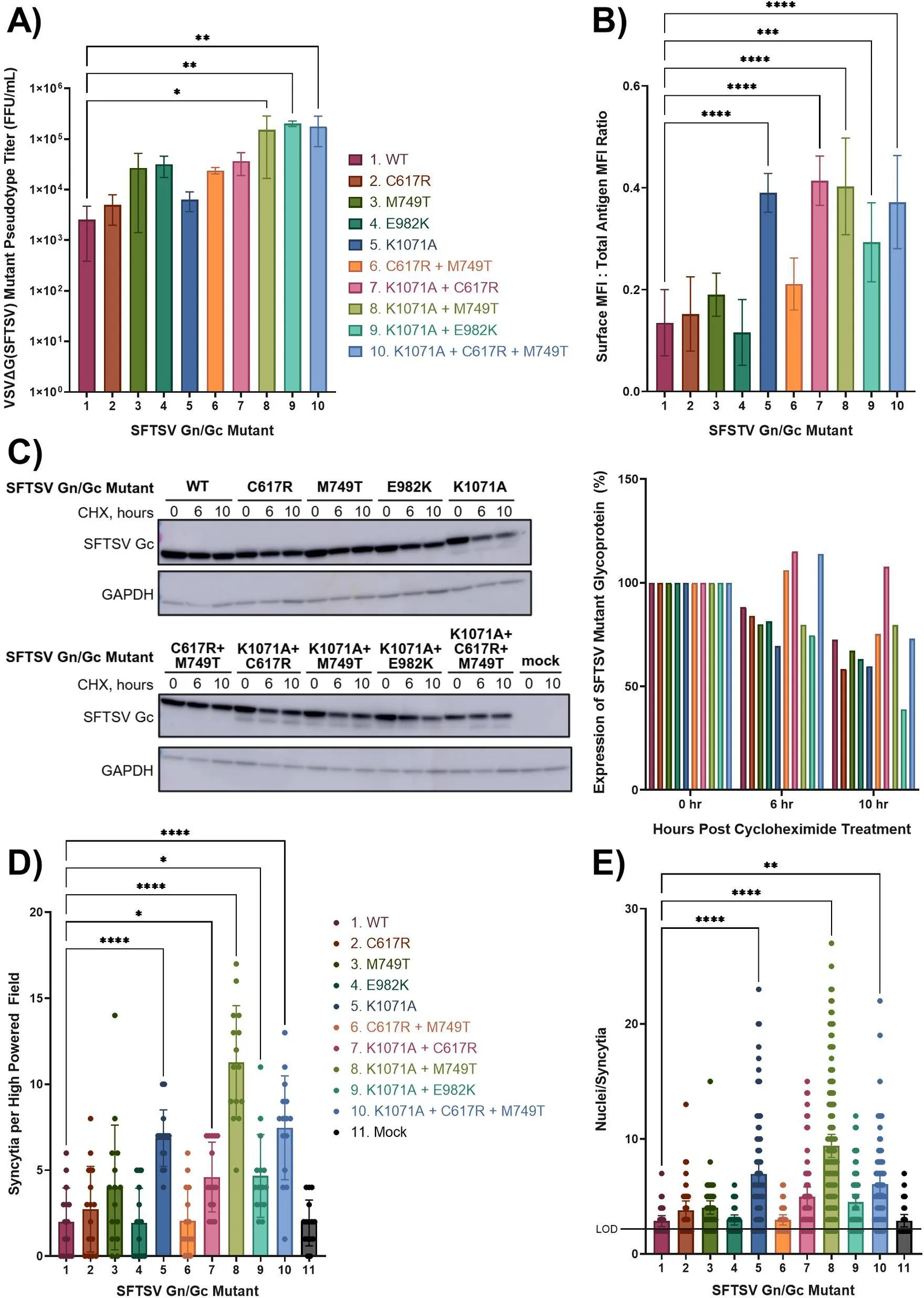
Mutations in SFTSV glycoprotein increase titers of VSVΔG pseudotypes via multiple mechanisms. (**A**) Titers of VSVΔG(SFTSV) mutant pseudotypes (FFU/mL) (ordinary one-way ANOVA with Dunnett’s multiple comparisons test; *, *P* < 0.05; **, *P* < 0.01). (**B**) Surface expression of SFTSV mutant glycoprotein (ratio of MFI of surface SFTSV antigen to MFI of total antigen) (ordinary one-way ANOVA with Dunnett’s multiple comparisons test; ***, *P* < 0.001; ****, *P* < 0.0001). (**C**) Stability of SFTSV mutant glycoproteins treated with 50 μg/mL cycloheximide or DMSO as vehicle control and evaluated 6 h and 10 h post-treatment. Protein expression in cells treated with cycloheximide was quantified via ImageJ and reported as a percentage of expression in DMSO-treated cells. (**D**) Syncytia per high-powered field and (**E**) nuclei per syncytia in cells transfected with SFTSV mutants and transiently treated with 50 mM MES-buffered PBS at pH 5.5 (ordinary one-way ANOVA with Dunnett’s multiple comparisons test; *, *P* < 0.05; **, *P* < 0.01; ****, *P* < 0.0001; LOD: limit of detection, two nuclei).

rVSV-SFTSV viruses are attenuated in cell culture, but serial passage of rVSV-SFTSV results in the spontaneous acquisition of mutations in the SFTSV glycoprotein that may improve viral replication (63, 64, 107). To evaluate the impact of the mutations C617R, M749T, E982K, and K1071A on incorporation of the SFTSV glycoprotein into VSV particles, we developed CMV promoter plasmids encoding a codon-optimized coding region of the M segment of SFTSV and used mutagenesis to generate plasmids incorporating each mutation alone and in combination. These plasmids were used to produce VSVΔG(SFTSV) pseudotypes incorporating each mutant SFTSV glycoprotein (Fig. 1A). VSVΔG pseudotypes do not encode a glycoprotein, and for these studies, fluorescent RFP is inserted in place of VSV-G. Though VSVΔG does not encode its own glycoprotein, budding VSVΔG particles can incorporate foreign viral glycoproteins expressed *in trans* through transfected or stably expressed genes. These virions enter cells using the biology of the incorporated glycoprotein they harbor but cannot produce infectious progeny in the target cell. Since these virions are only capable of a single round of replication, they are useful tools in studying various aspects of virion assembly without multiple rounds of replication yielding confounding results (111, 117). Recovery of all SFTSV mutant pseudotypes indicates successful incorporation of each mutant glycoprotein onto VSV particles. We show that each substitution alone slightly increases the VSVΔG(SFTSV) titer, with E928K and M749T having the largest impact of single substitutions, resulting in an increase in titer of approximately one log. More importantly, synergism between substitutions is also evident, particularly with the inclusion of K1071A with E982K or M749T, resulting in a 95-fold increase over the WT titers (Fig. 1A).

As mentioned, VSVΔG pseudotypes are created when a budding VSV virion acquires a foreign viral glycoprotein, typically expressed on the plasma membrane due to the site of VSV assembly (111). However, SFTSV glycoproteins are normally retained in the ERGIC where SFTSV particles assemble and bud (118). We hypothesized that the substitutions we observed may have a role in redistributing SFTSV glycoproteins to the surface of the cell, resulting in increased availability for incorporation into pseudotypes. To investigate this, we used flow cytometry to compare the expression of SFTSV glycoproteins on the surface of cells to SFTSV glycoproteins maintained in intracellular compartments (Fig. 1B). This was done by staining surface proteins with mouse polyclonal serum reactive to SFTSV glycoproteins prior to permeabilization and labeling of internal proteins with rabbit polyclonal antibodies specific to SFTSV Gn and Gc. This allowed us to directly compare localization of the glycoproteins within individual cells. We found that K1071A has a large impact on surface expression, increasing the ratio of surface-expressed protein to internally retained protein nearly four times over the WT glycoprotein. However, we did not see any further increase in surface expression with the addition of other substitutions. Given the phenotypes seen with the VSVΔG pseudotypes, it is surprising that no synergism is seen with the substitutions in terms of surface expression, and this suggests that there may be additional mechanisms responsible for the increased pseudotype titers.

To account for the effects of the substitutions that do not alter surface expression, we hypothesized that altered surface trafficking of the K1071A variant might result in exposure of SFTSV glycoproteins to low pH within the secretory pathway or to endocytosis and subsequent degradation from the cell membrane (119, 120). The additional substitutions might serve to increase the stability of the K1071A protein, resulting in increased incorporation onto VSV particles and increased pseudotype titers. To assess SFTSV protein stability, we transfected cells with the mutant glycoproteins and treated the cells with DMSO or cycloheximide, which causes translation arrest. We harvested the cells 6 h or 10 h post-treatment. Proteins were analyzed via Western blot using GAPDH as a loading control (Fig. 1C). We found that glycoproteins with the K1071A substitution were much less stable, declining to approximately 52% protein expression at 10 h post-treatment compared to 79% protein expression with the WT glycoprotein. The addition of substitutions to K1071A, particularly K1071A+C617R, seemed to increase the stability of the glycoprotein.

All the mutations identified are in the Gc component of the SFTSV glycoprotein, which is responsible for fusion and entry into cells (39–43). Given this, we next hypothesized that the substitutions might affect pseudotype infectivity by impacting the fusogenicity of the glycoprotein. To evaluate this, we transfected cells with the mutant SFTSV glycoproteins and transiently treated the cells with an acidic buffer (pH 5.5) 48 h post-transfection. After low pH treatment, the cells were allowed to fuse for 4 h before quantifying fusion by counting the number of syncytia in five high-powered fields per condition (Fig. 1D) and the number of nuclei per syncytium (Fig. 1E). Representative images are included in Fig. S1. As one might expect with an increase in surface expression, we saw that glycoproteins bearing the K1071A substitution resulted in overall increases in syncytia and the number of nuclei within. We saw further increases with K1071A+M749T, and this additional substitution resulted in the formation of an increased number and size of syncytia compared to K1071A alone. This indicates that increases in fusogenicity may play a role in the increased pseudotype titers. Overall, we find that substitutions in the SFTSV glycoprotein increase VSVΔG(SFTSV) pseudotype titers via multiple mechanisms.

### Effect of K1071A mutation on SFTSV glycoprotein surface expression

The protein localization phenotype resulting from K1071A is striking, given that most bunyaviral glycoproteins are directed to internal compartments prior to budding (84–91). Gn/Gc complexes encoded by most bunyaviruses localize to the Golgi when expressed separately from other viral factors (84, 86, 87, 90, 91, 93, 94, 121–123). The K1071A mutation is located in the cytoplasmic tail of the Gc component of the SFTSV glycoprotein, a region in the protein that has been implicated in ER targeting in other bunyaviruses (94). More specifically, the K1071A substitution is located three amino acids from the carboxy terminus of the protein, otherwise referred to as the −3 position. This residue in the −3 position is accompanied by arginine, an additional positively charged amino acid, in the −5 position. This motif, RxKxx, resembles the canonical COPI-binding signal KxKxx. COPI is a complex of several proteins that functions to recycle cargo from the Golgi to the ER and retain cargo within the ER, among other things. Cargo-binding components of COPI typically interact with target proteins bearing dilysine motifs, either KKxx or KxKxx, that bind to α-COP or β′-COP, respectively. Noncanonical β′-COP targeting sequences, including KxHxx, have been identified in the cytoplasmic tails of other viruses (96, 100, 104, 124–126). We hypothesized that the K1071A substitution inhibits interaction with β′-COP via abrogation of a non-canonical RxKxx motif, resulting in increased surface expression.

**Fig 2:**
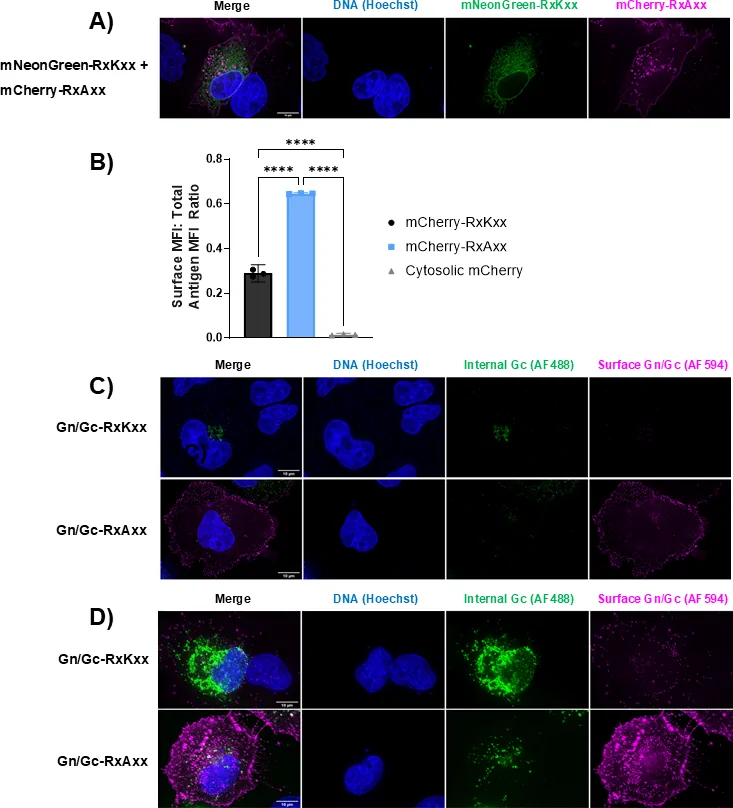
K1071A substitution redistributes SFTSV Gn and Gc from intracellular compartments to the cell surface. (**A**) Fluorescence microscopy of cells co-expressing mNeonGreen-RxKxx and mCherry-RxAxx from separate plasmids. (**B**) Surface expression of mCherry (ratio of MFI of surface mCherry to MFI of total mCherry antigen) (ordinary one-way ANOVA with Tukey’s multiple comparisons test; ****, *P* < 0.0001). Data are representative of three independent experiments. (**C**) Immunofluorescence microscopy of internal (permeabilized) and surface antigen in transfected A549 cells expressing wild-type (RxKxx) or K1071A mutant (RxAxx) SFTSV Gn/Gc. (**D**) Immunofluorescence microscopy of internal (permeabilized) and surface antigens in transfected U2OS cells expressing wild-type (RxKxx) or K1071A mutant (RxAxx) SFTSV Gn/Gc. Images in panels A, C, and D were collected at 100× under oil immersion, and scale bars are 10 μm in length. Images are of representative equatorial slices.

To dissect the impact of the cytoplasmic tail mutation (K1071A) on Gc protein localization, we generated localization reporters consisting of SFTSV glycoprotein chimeras in which Gc ectodomain sequences were replaced with fluorescent reporters (mCherry or mNeonGreen). A549 cells were cotransfected with a plasmid encoding mNeonGreen appended to the WT Gc transmembrane domain and cytoplasmic tail (mNeonGreen-RxKxx) and a plasmid encoding mCherry appended to the transmembrane domain and cytoplasmic tail bearing the K1071A substitution (mCherry-RxAxx) (Fig. 2A). In agreement with results from flow cytometry experiments (Fig. 1B), mNeonGreen-RxKxx exhibited an ER distribution pattern with undetectable levels at the cell surface. In contrast, mCherry-RxAxx was clearly detectable at the cell surface (Fig. 2A). This suggests that the RxKxx motif alone is sufficient to distribute proteins to the ER, regardless of the identity of the ectodomain, and that mutation of the −3 lysine abrogates ER retention. To quantify the phenotype exhibited in Fig. 2A, we transfected cells with mCherry-RxKxx, mCherry-RxAxx, or cytosolic mCherry to compare the surface expression of mCherry via flow cytometry. Non-permeabilized cells were stained with an antibody specific to mCherry and labeled with Alexa Fluor 488 to differentiate surface antigen from the intracellular mCherry signal. The percentage of total mCherry protein that was expressed on the surface of cells is reported in Fig. 2B. As expected from the IF results, mCherry-RxKxx was predominantly expressed inside the cell, and only a small percentage (less than 30%) of the total protein was expressed at the cell surface. In accordance with previous results, mCherry-RxAxx exhibited significantly more cell surface expression, with greater than 64% of proteins trafficking to the cell surface. This represents a greater than twofold increase in cell surface expression when comparing a construct with the Gc WT transmembrane domain and cytoplasmic tail to one with the −3 lysine-to-alanine mutation (Fig. 2B).

Evidence that Gn dictates Golgi targeting includes the observations that Gn expressed in isolation of Gc localizes to the Golgi, and recruitment of Gc from the ER to the Golgi requires co-expression with Gn. The contribution made by Gc to the overall distribution of Gn/Gc complexes has been less well studied and remains unclear (93, 94). To study the effect of the K1071A substitution on the localization of the Gn/Gc complex, A549 cells were transfected with expression plasmids encoding SFTSV Gn/Gc with the WT tail sequence (Gn/Gc-RxKxx) or Gn/Gc with the K1071A substitution (Gn/Gc-RxAxx). Similar to the methods used for Fig. 1B, external proteins were labeled with mouse polyclonal serum reactive to SFTSV glycoproteins, and internal proteins were labeled with rabbit polyclonal antibodies specific for SFTSV Gn and Gc, and cells were imaged by immunofluorescence microscopy. Surface signal from cells expressing Gn/Gc-RxKxx was low or absent in most antigen-positive cells (Fig. 2C). Internal signal in most cells was concentrated in a perinuclear region that was likely the Golgi. In contrast, surface signal intensity of cells transfected with Gn/Gc-RxAxx was significantly greater, and full cell boundaries were visible. Gn/Gc-RxAxx also distributed differently within intracellular compartments. Given the decreased stability of proteins with the K1071A substitution (Fig. 1C), it is possible that the proteins are endocytosed from the cell surface and targeted for lysosomal degradation, which may partially explain the altered intracellular distribution. Expression in a different cell line, U2OS, displayed similar staining patterns for Gn/Gc-RxKxx and Gn/Gc-RxAxx to those observed in A549 cells; however, the surface localization of the RxAxx mutant is more obvious (Fig. 2D). Taken together, these data suggest that the K1071A substitution redistributes Gn/Gc complexes from intracellular compartments to the cell surface.

**Fig 3:**
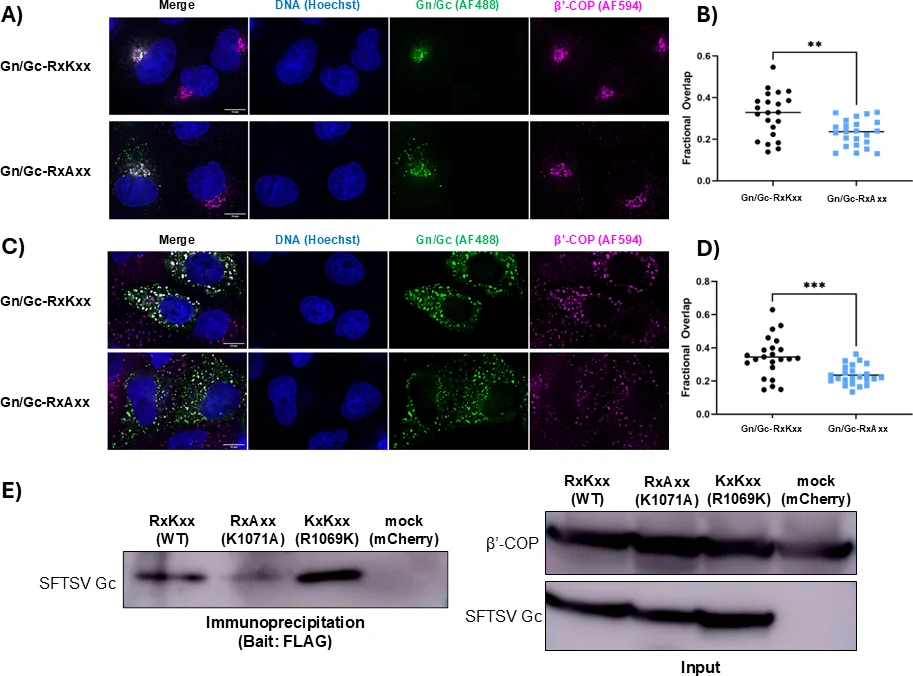
K1071A mutation increases surface expression of SFTSV glycoproteins via inhibition of COPI binding. (**A**) Immunofluorescence microscopy of vehicle (DMSO)-treated A549 cells transiently expressing SFTSV Gn/Gc-RxKxx or Gn/Gc-RxAxx. Cells were stained for the Golgi marker β′-COP. Images were acquired at 100x magnification. Images are representative equatorial slices. Scale bars are 10 μm in length. (**B**) Fractional overlap of Gn/Gc-RxKxx or Gn/Gc-RxAxx and β′-COP. Dots represent single cells (*n* = 22 cells/condition) pooled from two independent experiments (Student’s *t*-test; **, *P* < 0.01). (**C**) Immunofluorescence microscopy of A549 cells transfected to express SFTSV Gn/Gc-RxKxx or Gn/Gc-RxAxx and pretreated for 3 h with 33 μM nocodazole, then stained for endogenous β′-COP. Images were acquired at 100x magnification. Images are of representative equatorial slices. Scale bars are 10 μm in length. (**D**) Fractional overlap of Gn/Gc-RxKxx or Gn/Gc-RxAxx with β′-COP. Each dot represents an individual cell with *n* = 23 cells/condition collected from two independent experiments (Student’s *t*-test; ***, *P* < 0.001). (**E**) Immunoprecipitation of SFTSV Gn/Gc-RxKxx, -RxAxx, or -KxKxx (positive control) from 293T cells cotransfected with SFTSV Gn/Gc-RxKxx, -RxAxx, or -KxKxx and FLAG-tagged β′-COP. Protein was bound to magnetic beads conjugated to an anti-FLAG antibody, washed thoroughly with cell lysis buffer, and eluted by boiling. Proteins recovered from IP were evaluated via SDS-PAGE and Western blot using an antibody against SFTSV Gc and an antibody against β′-COP. Data are representative of four independent experiments.

Given the significant impact of the K1071A substitution on protein trafficking and surface expression of the SFTSV glycoprotein, and our hypothesis that the K1071A substitution altered a potential noncanonical COPI binding site, we wanted to interrogate the mechanism responsible for that phenotype more thoroughly. We used transient SFTSV Gn/Gc expression to address, via immunofluorescence microscopy, whether the viral glycoproteins colocalize with endogenous β′-COP. A549 cells were transfected with the WT plasmid (Gn/Gc-RxKxx) or the K1071A plasmid (Gn/Gc-RxAxx) and stained with antibodies against SFTSV Gn/Gc and β′-COP. As a component of COPI, which is involved in retrograde trafficking within the Golgi cisternae and between the Golgi and ER, β′-COP is localized primarily to the Golgi in mammalian cells (127, 128). Treatment of cells with the microtubule depolymerizing agent nocodazole disrupts Golgi structure, relocalizes Golgi resident proteins into dispersed cytoplasmic vesicles, and allows improved spatial resolution between the Golgi and adjacent structures (129). In cells treated with vehicle, β′-COP staining was most intense in a perinuclear region consistent with the Golgi (Fig. 3A). Gn/Gc-RxKxx and Gn/Gc-RxAxx signals overlapped with that of β′-COP, but Gn/Gc-RxAxx appeared more dispersed throughout the cytosol. Similar results were seen in cells transiently expressing Gn/Gc-RxKxx or Gn/Gc-RxAxx that were treated with nocodazole before fixation and staining for viral antigen and β′-COP (Fig. 3C). The fractional overlap between Gn/Gc-RxAxx and β′-COP was reduced compared to that between Gn/Gc-RxKxx and β′-COP under both mock (Fig. 3B) and nocodazole-treated conditions (Fig. 3D). These results suggest that the fraction of Gn/Gc-RxAxx retained in the Golgi is reduced compared to Gn/Gc-RxKxx.

Finally, to directly address the hypothesis that the K1071A substitution increases surface expression via inhibition of binding to β′-COP, we evaluated binding of SFTSV Gn/Gc-RxKxx or Gn/Gc-RxAxx to β′-COP via an immunoprecipitation assay. We cotransfected HEK293T cells with Gn/Gc-RxKxx or Gn/Gc-RxAxx expression plasmids along with a commercially available β′-COP plasmid tagged with FLAG at the carboxy terminus. This commercially available β′-COP plasmid was chosen based on reports that a C-terminal-tagged β′-COP is capable of binding cargo (130, 131). An expression plasmid with an R1069K substitution (Gn/Gc-KxKxx) was used as a positive control, as we expected that a canonical COPI-binding motif in the cytoplasmic tail would bind to β′-COP more efficiently. A plasmid expressing mCherry was used as a negative control. We harvested cell lysate 48 h post-transfection and precipitated proteins using anti-FLAG beads. The beads were thoroughly washed using cell lysis buffer, and bound protein was eluted by boiling. We found that Gn/Gc carrying the wild-type RxKxx motif co-precipitates with β′-COP. As anticipated, we found that Gn/Gc with a canonical binding motif, KxKxx, co-precipitates with β′-COP with the highest efficiency of the proteins tested. Very little immunoprecipitation of the mutant Gn/Gc-RxAxx protein was seen, which supports our hypothesis that the K1071A substitution inhibits binding to β′-COP (Fig. 3E).

### Characterization of replication-competent rVSV-SFTSV mutants

Next, we wanted to understand the effects of the mutations within the glycoprotein on replication-competent rVSV-SFTSV. Using published VSV launch strategies (109), we attempted to launch rVSV-SFTSV viruses bearing all the substitution combinations seen in Fig. 1. Despite numerous attempts, we were only able to launch a subset of these viruses: rVSV-SFTSV K1071A, rVSV-SFTSV K1071A+C617R, rVSV-SFTSV K1071A+M749T, and rVSV-SFTSV M749T. We were unable to launch rVSV-SFTSV bearing the WT glycoprotein that contains a non-canonical COPI-binding motif. Our lab first launched rVSV-SFTSV in 2013 for use in identifying SFTSV entry factors (44). Sequencing of the earliest stocks of this virus revealed that the K1071 residue was mutated, suggesting that this substitution may have been required for launch or effective replication after launch.

**Fig 4:**
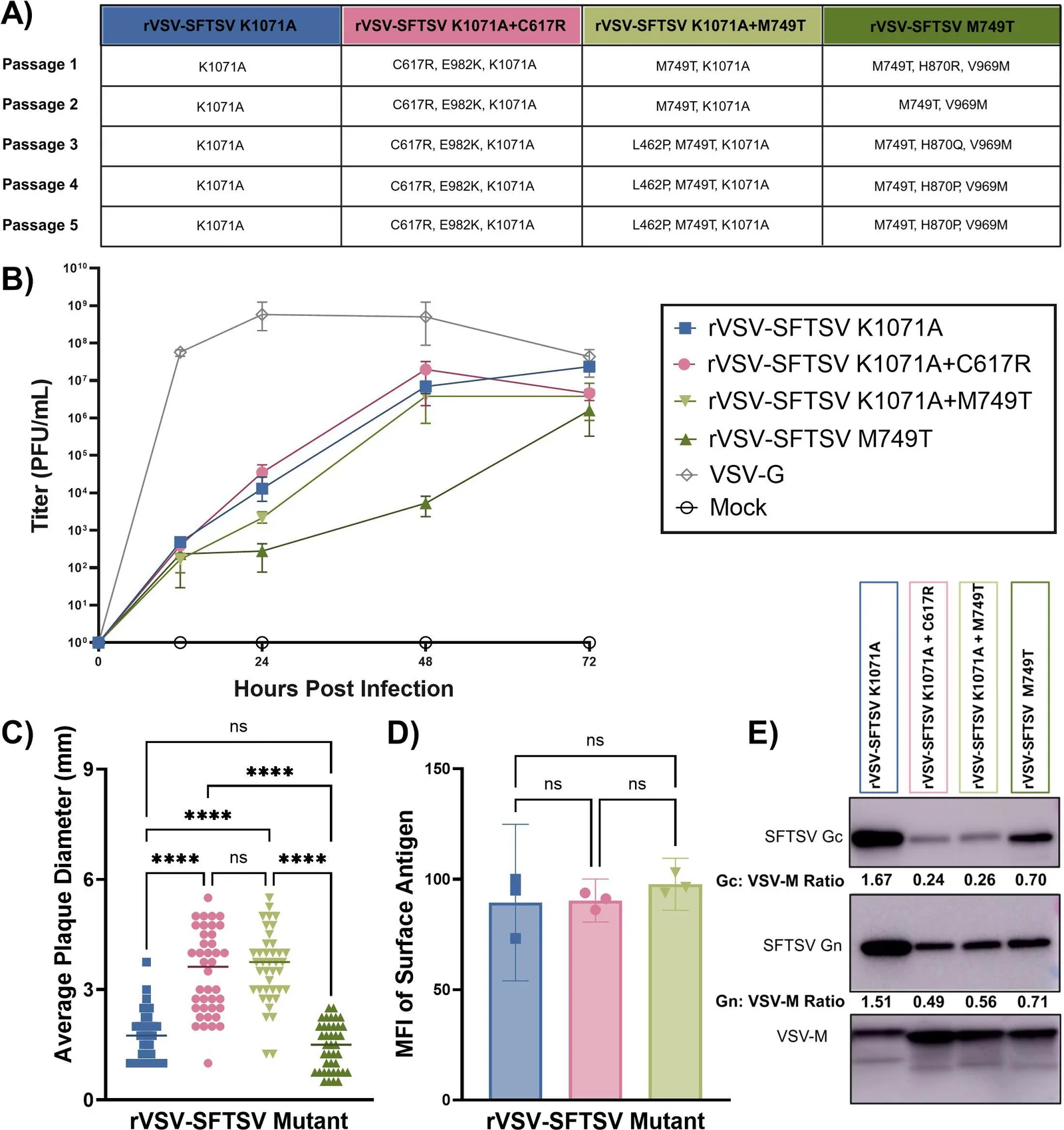
K1071A mutation enhances replication of rVSV-SFTSV and improves incorporation of SFTSV onto VSV virions. (**A**) Five passages of each rVSV-SFTSV mutant were sequenced via nanopore sequencing to assess genetic stability of the SFTSV glycoprotein. Nonsynonymous mutations were reported. (**B**) Replication kinetics of rVSV-SFTSV mutants compared to VSV-G. Vero E6 cells were infected at an MOI of 0.001, and samples of supernatant were collected at 12, 24, 48, and 72 h post-infection. Viral titers were evaluated via plaque assays on Vero E6 cells. (**C**) Plaques from each rVSV-SFTSV mutant were measured with a standard ruler and rounded to the nearest quarter of a millimeter (ordinary one-way ANOVA with Tukey’s multiple comparisons test; ****, *P* < 0.0001). (**D**) Surface expression of mutant glycoproteins in A549 cells infected with rVSV-SFTSV mutants at an MOI of 2. Cells were fixed and stained for flow cytometry 24 h post-infection (ordinary one-way ANOVA with Tukey’s multiple comparisons test; no significant differences found). These data are representative of three independent experiments. (**E**) Incorporation of mutant glycoproteins onto rVSV particles. Vero E6 cells were seeded in 15 cm plates and infected with each rVSV-SFTSV mutant at an MOI of 0.001. After 48 h of infection, supernatant was harvested, and virions were purified via ultracentrifugation through a sucrose cushion at 26,000 × *g* for 2 h. Virions were lysed using Triton X-100, and protein samples were evaluated via SDS-PAGE and Western blot using antibodies specific to SFTSV Gn, Gc, and VSV-M. Ratios of Gn or Gc to VSV-M were calculated using ImageJ. These data are representative of two independent experiments.

Following launch of rVSV-SFTSV K1071A, rVSV-SFTSV K1071A+C617R, rVSV-SFTSV K1071A+M749T, and rVSV-SFTSV M749T, we passaged each virus five times and sequenced the SFTSV glycoprotein in each passage to determine whether the mutations were genetically stable. We found that rVSV-SFTSV K1071A was stable across five passages, but each other viral mutant acquired additional mutations. Surprisingly, rVSV-SFTSV K1071A+C617R acquired the E982K substitution in the first passage after launch. rVSV-SFTSV K1071A+M749T acquired a substitution in the transmembrane domain of Gn, L462P, by passage 3. rVSV-SFTSV M749T was the least stable of the viruses, picking up numerous mutations that changed between passages (Fig. 4A). For consistency and brevity, we will continue to refer to each virus with the mutations that were present at launch, not the mutations that arose spontaneously over passage.

To determine whether the mutations impacted the replication of rVSV-SFTSV, we infected Vero E6 cells with the rVSV-SFTSV mutants at a low MOI and collected virus-containing supernatant at 12, 24, 48, and 72 h post-infection. We used standard plaque assays to evaluate the titer over the course of the replication experiment, and titers were compared to VSV with the native VSV-G glycoprotein. All the rVSV-SFTSV viruses replicated to high titers that would be appropriate for large-scale experiments, but they were still highly attenuated compared to VSV. All rVSV-SFTSV variants bearing the K1071A substitution reached a maximum titer of approximately 10^7^ PFU/mL, but rVSV-SFTSV M749T reached a peak titer of 10^6^ PFU/mL. All the viruses bearing the K1071A substitution replicated significantly more rapidly than the virus with only the M749T substitution, but we were surprised to find that there was no significant difference in replication between rVSV-SFTSV K1071A, rVSV-SFTSV K1071A+C617R, and rVSV-SFTSV K1071A+M749T (Fig. 4B).

The plaque assays used to calculate the titers also revealed an interesting phenotype in plaque sizes in Vero E6 cells. All rVSV-SFTSV viruses produced distinguishable plaques 96 h post-infection. However, there is a significant difference in the size of plaques at this time point. rVSV-SFTSV K1071A plaques are small, with an average diameter of 1.7 mm. The plaques for the K1071A+C617R and K1071A+M749T viruses are significantly larger and have an average diameter of 3.5 and 3.6 mm, respectively (Fig. 4C). This may reflect increased cell-cell spread, potentially resulting from increased fusogenicity of the glycoproteins. Supporting this idea, we previously demonstrated increased fusogenicity of the K1071A+M749T glycoprotein mutant (Fig. 1D and E). The recombinant virus carrying the M749T variant produced small plaques that were comparable to rVSV-SFTSV K1071A (Fig. 4C).

Although our flow cytometry data exploring the surface expression of the mutant glycoprotein plasmids suggested that there was not a significant difference in surface localization in glycoproteins bearing the K1071A substitution (Fig. 1B), we wanted to rule this out as an explanation for the differences in plaque sizes. We infected A549 cells at an MOI of 2 and evaluated surface glycoprotein expression in infected cells by flow cytometry. We excluded rVSV-SFTSV M749T from this analysis due to its slow replication, which prevented analysis of surface expression at the same time point as the other viruses. As expected, we found that there was no significant difference in surface expression between the K1071A, K1071A+C617R, and K1071A+M749T recombinant viruses (Fig. 4D). This suggests that the differences in plaque sizes result from a mechanism independent of surface expression.

Finally, we evaluated incorporation of the glycoproteins into purified rVSV virions via a Western blot comparing expression of SFTSV Gn and Gc to VSV-M. We found that rVSV-SFTSV K1071A Gn and Gc were incorporated much more efficiently than any of the other mutants, with an expression of 1.51 and 1.67 times VSV-M, respectively. Unexpectedly, Gc of rVSV-SFTSV K1071A+C617R and rVSV-SFTSV K1071A+M749T were not efficiently incorporated, with an expression of approximately 0.25 times VSV-M for both viruses (Fig. 4E).

### Characterization of replication-competent rVSV-HRTV mutants

We next sought to determine whether the mutations identified in SFTSV could be relevant to other viruses in the *Bandavirus* genus. HRTV is a closely related bandavirus with a high case fatality rate, and no vaccines have yet been developed to prevent infection (31). We wanted to develop an rVSV-HRTV vaccine for preclinical testing, and we hypothesized that the SFTSV substitutions might improve replication of the virus, resulting in improved ease of production and enhanced immunogenicity. Notably, HRTV carries a non-canonical COPI-binding motif RxKxx, identical to the one in SFTSV.

Based on our initial data from the VSVΔG(SFTSV) pseudotypes (Fig. 1A) and surface expression (Fig. 1B), we decided to focus on the K1071A substitution and the combination of K1071A+C617R+M749T substitutions. Using sequence homology, we built these substitutions into the cognate positions of the HRTV glycoprotein. For HRTV, this corresponded to K1074, C621, and V753. For K1074 and V753, we made the same substitutions used in SFTSV: K1074A and V753T. However, we mutated C621 to a serine based on published data that showed a C621S substitution occurred spontaneously in HRTV passaged in mice (108). Since this substitution seemed to improve replication of HRTV in animals, we hypothesized that it would also be beneficial in rVSV-HRTV.

**Fig 5:**
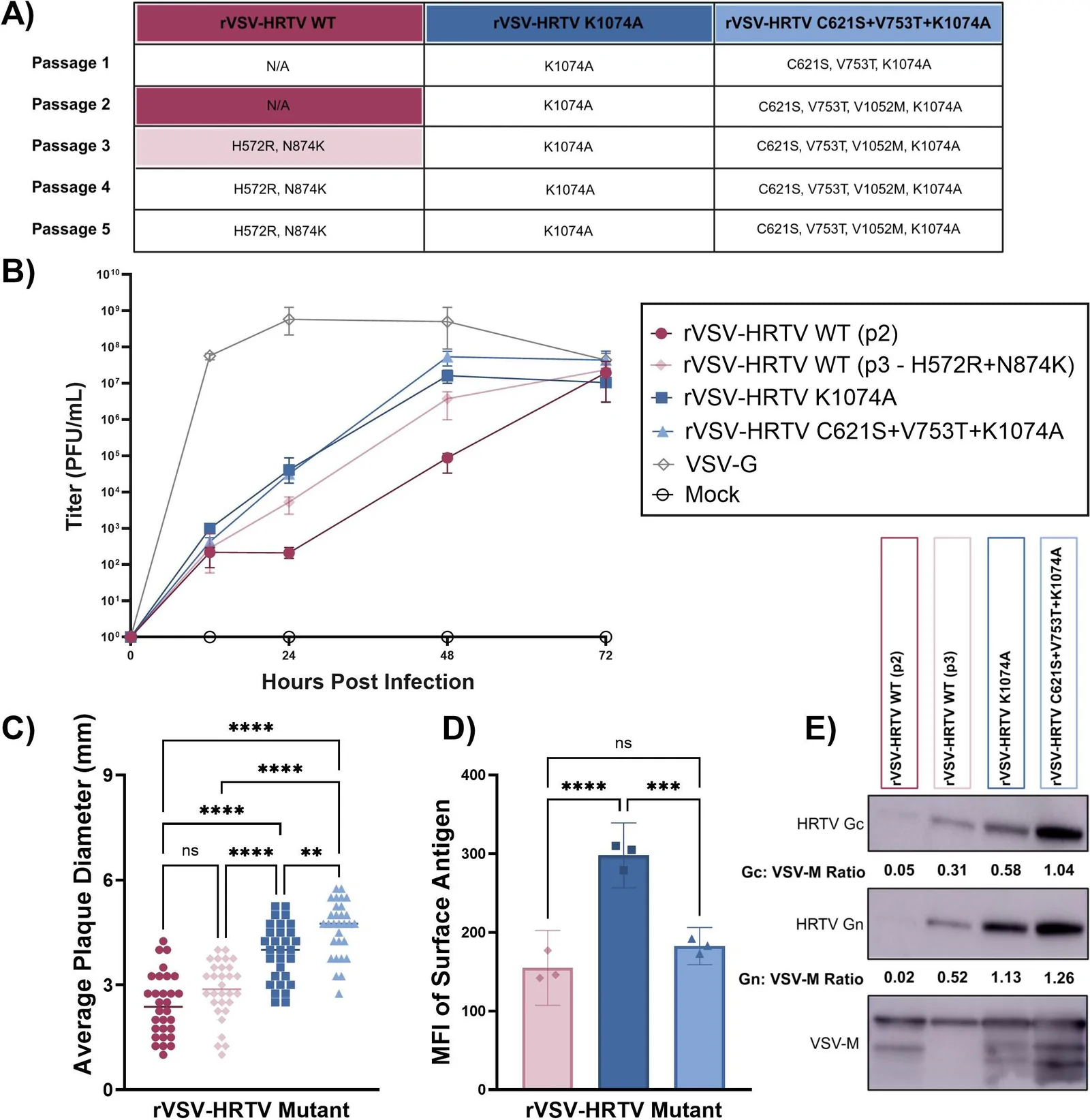
K1074A mutation is also effective in rVSV-HRTV, leading to enhanced replication and glycoprotein expression on the surface of infected cells. (**A**) Five passages of each rVSV-HRTV mutant were sequenced via nanopore sequencing to assess genetic stability of the HRTV glycoprotein. Nonsynonymous mutations are reported. (**B**) Replication kinetics of rVSV-HRTV mutants compared to VSV-G. Vero E6 cells were infected at an MOI of 0.001, and samples of supernatant were collected at 12, 24, 48, and 72 h post-infection. Viral titers were evaluated via plaque assays on Vero E6 cells. (**C**) Plaques from each rVSV-HRTV mutant were measured with a standard ruler and rounded to the nearest quarter of a millimeter (ordinary one-way ANOVA with Tukey’s multiple comparisons test; **, *P* < 0.01; ****, *P* < 0.0001). (**D**) Surface expression of mutant glycoproteins in A549 cells infected with rVSV-HRTV mutants at an MOI of 2. Cells were fixed and stained for flow cytometry 24 h post-infection (ordinary one-way ANOVA with Tukey’s multiple comparisons test; ***, *P* < 0.001; ****, *P* < 0.0001). These data are representative of three independent experiments. (**E**) Incorporation of mutant glycoproteins onto rVSV particles. 15 cm plates of Vero E6 cells were infected with each rVSV-HRTV mutant at an MOI of 0.001. 48 h post-infection, supernatant was harvested, and virions were purified via ultracentrifugation through a sucrose cushion at 28,000 × *g* for 2 h. Virions were lysed using Triton X-100, and protein samples were evaluated via SDS-PAGE and Western blot using antibodies specific to VSV-M and SFTSV antibodies that cross-react with HRTV Gn and Gc. Ratios of Gn or Gc to VSV-M were calculated using ImageJ. These data are representative of two independent experiments.

Using the same rVSV launch protocol as described above, we attempted to launch rVSV-HRTV encoding the WT, K1074A, and C621S+V753T+K1074A variants. All these viruses were successfully rescued, which was a surprise to us, given the difficulty we had in recovering rVSV-SFTSV WT. We again sequenced the viruses to determine whether the genome was stable over multiple passages. We found that rVSV-HRTV WT was stable to passage 2 but acquired two additional mutations by passage 3, causing the changes H572R and N874K. rVSV-HRTV C621S+V753T+K1074A acquired one additional mutation by passage 2, a V1052M substitution. In contrast, rVSV-HRTV K1074A was stable through passage 5 (Fig. 5A).

Following launch of the viruses, we did a time-course experiment using the same methods as those used for Fig. 4B. For comparison, we included the second passage of rVSV-HRTV WT because sequencing showed that this passage had not acquired any additional mutations (Fig. 5A). Titers at each time point were again compared to those of VSV-G. We saw very similar replication kinetics between rVSV-SFTSV K1071A and rVSV-HRTV K1074A, with similar titers at each time point and peak titers reaching approximately 10^7^ PFU/mL. Again, we did not see a significant difference between rVSV-HRTV K1074A and rVSV-HRTV C621S+V753T+K1074A, suggesting that the K1074A substitution is sufficient to improve rVSV-HRTV replication, as was the case with rVSV-SFTSV. rVSV-HRTV WT (p2) replicated significantly slower than the viruses with the K1074A substitution, resulting in a difference in titer of approximately 2.5 logs at 24 h post-infection and 2 logs at 48 h post-infection. rVSV-HRTV WT (p3), which had acquired additional changes, grew with an intermediate phenotype, replicating faster than WT (p2) but slower than the viruses with the K1074A substitution (Fig. 5B). This suggests that there are multiple mutation pathways that the virus can take to improve replication *in vitro*.

We measured the sizes of plaques formed by each rVSV-HRTV mutant, and again rVSV-HRTV WT (p2) was included as a control. No significant difference in plaque sizes was seen between passages 2 and 3 of rVSV-HRTV WT, suggesting that the two additional mutations acquired spontaneously do not appear to impact the cell-cell spread of the virus. Plaques for rVSV-HRTV K1074A were significantly larger than either of the rVSV-HRTV WT passages, but rVSV-HRTV C621S+V753T+K1074A formed the largest plaques of any of the viruses (Fig. 5C). This may reflect that the additional mutations aid in fusogenicity and cell-cell spread, as was suggested by the rVSV-SFTSV results.

Next, we evaluated the cell surface expression of glycoproteins in cells infected with the rVSV-HRTV mutants by flow cytometry. We excluded rVSV-HRTV WT (p2) due to its slower replication, which prevented analysis at the same time point as the other viruses. As expected, we found a significant difference in surface expression between rVSV-HRTV WT (p3) and rVSV-HRTV K1074A, which indicates that the substitution of the lysine residue at the −3 position is responsible for redistribution of glycoproteins to the cell surface in HRTV as well as SFTSV. To our surprise, we found that the surface expression of rVSV-HRTV C621S+V753T+K1074A was significantly lower than rVSV-HRTV K1074A and was comparable to rVSV-HRTV WT (Fig. 5D). It is unclear why this would be the case, especially given the similarity in replication kinetics between rVSV-HRTV K1074A and rVSV-HRTV C621S+V753T+K1074A.

We also evaluated incorporation of HRTV Gn and Gc into VSV virions. Incorporation of Gn and Gc from HRTV WT (p2) was extremely inefficient, and these proteins were barely detectable in purified virions. The substitutions that arise spontaneously in rVSV-HRTV WT by passage 3, H572R and N874K, improve incorporation of the glycoproteins relative to passage 2. The K1074A substitution dramatically improves glycoprotein incorporation relative to rVSV-HRTV WT (p3), but the combination of C621S+V753T+K1074A substitutions results in the most efficient incorporation of HRTV glycoproteins into virions (Fig. 5E).

### Evaluation of rVSV-HRTV mutants as vaccine candidates

Our ultimate goal of this study was to identify mutations in rVSV-SFTSV and rVSV-HRTV that would be genetically stable, improve our ability to produce high levels of the viruses for clinical studies, and contribute to a more immunogenic vaccine candidate. We previously published work on rVSV-SFTSV, where we showed that a single dose of an rVSV-SFTSV vaccine with the K1071E and E982K substitutions was protective against lethal SFTSV challenge and also provided cross-protection against HRTV (64, 112). To determine whether substitutions that affect HRTV glycoprotein function *in vitro* and *in vivo* replication would also improve the efficacy of an rVSV-HRTV vaccine candidate, we characterized our rVSV-HRTV mutants *in vivo* and evaluated safety, immunogenicity, and protection against lethal challenge.

**Fig 6:**
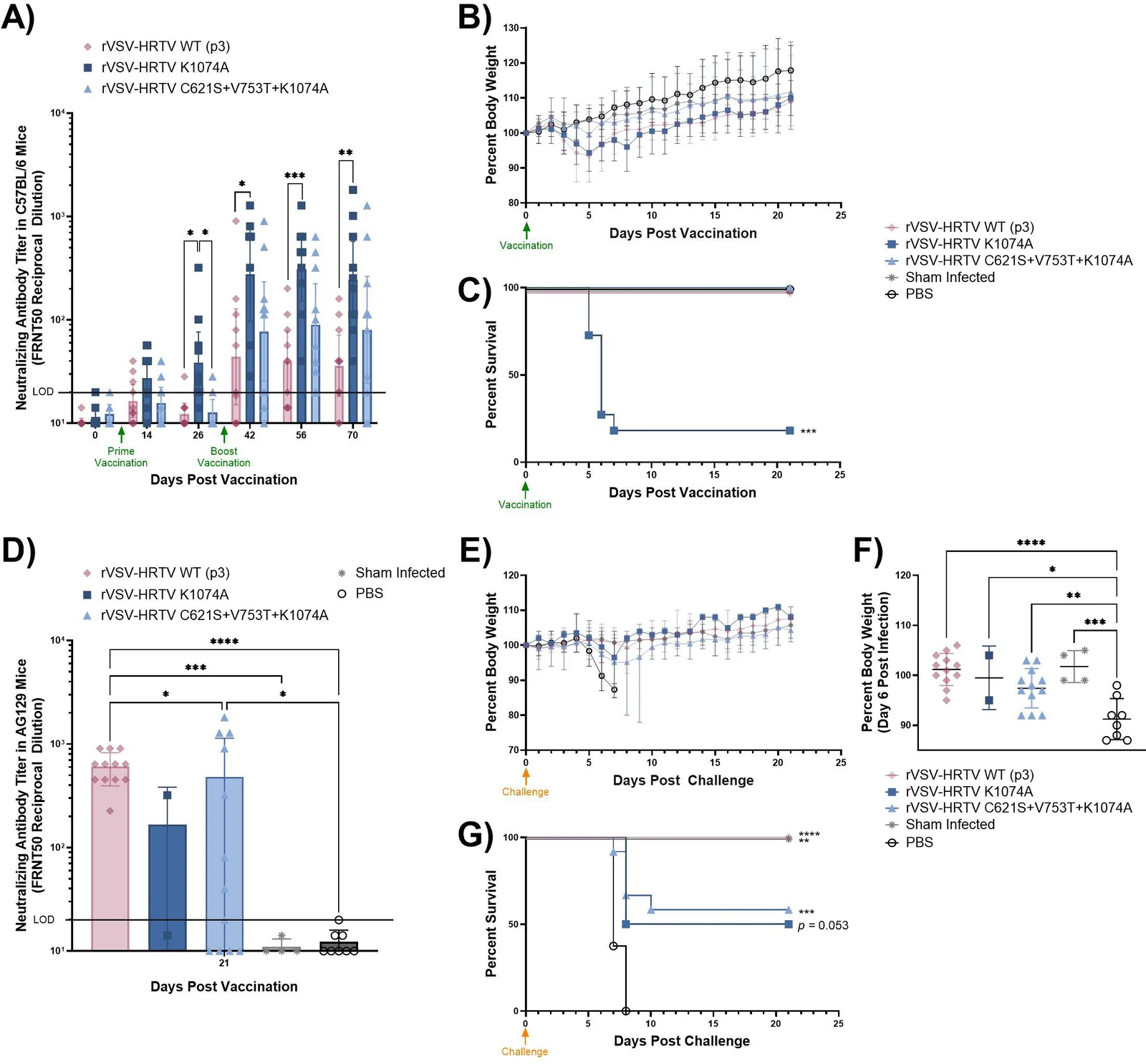
rVSV-HRTV K1074A induces higher neutralizing antibody titers in immunocompetent mice and exhibits enhanced replication *in vivo* in immunocompromised mice. (**A**) Five male and five female C57BL/6 mice were IP immunized with 10^6^ PFU of rVSV-HRTV WT (p3), K1074A, or C621S+V753T+K1074A at day 0 and day 28. Sera were collected from animals prior to vaccination (day 0), and at 14, 26, 42, 56, and 70 days post-vaccination. Sera were evaluated for neutralizing antibodies against HRTV via FRNT_50_. Horizontal line indicates the limit of detection (LOD) of the assay (20) (two-way ANOVA with Tukey’s multiple comparisons test performed on log-normalized data; *, *P* < 0.05; **, *P* < 0.01; ***, *P* < 0.001). (**B and C**) Six male and six female AG129 mice were IP injected with 10^2^ PFU of rVSV-HRTV vaccine candidates. (**B**) Weight loss (mean and range) and (**C**) survival curves are shown for 21 days following injection. Statistical significance of survival was evaluated via the Mantel-Cox log-rank test compared to mice vaccinated with PBS placebo (***, *P* < 0.0001). (**D**) Blood was collected from AG129 mice that survived vaccination with 10^2^ PFU of rVSV-HRTV WT (p3), K1074A, or C621S+V753T+K1074A, and neutralizing antibodies against HRTV on day 21 post-vaccination were quantified via FRNT_50_. Horizontal line indicates LOD (20) of the assay (lognormal ordinary one-way ANOVA with Tukey’s multiple comparisons test; *, *P* < 0.05; ***, *P* < 0.001; ****, *P* < 0.0001). Surviving mice were challenged with MA-HRTV on day 23 post-vaccination. (**E**) Weight loss (mean and range) is shown for 21 days post-HRTV challenge of immunized mice, and (**F**) compared to the PBS vaccine placebo group at day 6 post-challenge (ordinary one-way ANOVA with Dunnett’s multiple comparisons test; *, *P* < 0.05; **, *P* < 0.01; ***, *P* < 0.001; ****, *P* < 0.0001). (**G**) Survival is shown for 21 days post-HRTV challenge (statistical significance of survival was evaluated via the Mantel-Cox log-rank test compared to mice vaccinated with PBS placebo; *, *P* < 0.05; **, *P* < 0.01; ***, *P* < 0.001; ****, *P* < 0.0001).

First, we vaccinated groups of 8-week-old C57BL/6 mice with two doses (10^6^ PFU each) of each virus via IP injections. For this experiment, five male and five female mice were included in each group. Mice were given the prime vaccination dose on day 0 and boosted on day 28. Mice were bled via the facial vein prior to vaccination (day 0), and at days 14, 26, 42, 56, and 70 post-prime vaccination, and serum was isolated and stored for analysis. We employed focus reduction neutralization tests to define the dilution at which 50% of a VSVΔG(HRTV) pseudovirus was neutralized by the serum (FRNT_50_). Our FRNT50 tests were shown to specifically identify anti-HRTV neutralizing antibodies, as serum from rVSV-ZEBOV-vaccinated mice was unable to neutralize VSVΔG(HRTV) pseudovirus, and serum from mice vaccinated with our rVSV-HRTV variants was unable to neutralize VSVΔG pseudovirus with the WT G glycoprotein (Fig. S2). We found that neutralizing antibody titers in these immunocompetent C57BL/6 mice were relatively low across all groups prior to the boost. However, rVSV-HRTV K1074A vaccination induced significantly higher titers than rVSV-HRTV WT (p3) and rVSV-HRTV C621S+V753T+K1074A at day 26 post-vaccination. Following the boost vaccination at day 28, we saw an increase in neutralizing titers in all vaccine groups. At each time point following the boost, rVSV-HRTV K1074A outperformed rVSV-HRTV WT (p3) by inducing significantly higher neutralizing antibody titers. Post-boost neutralizing antibody titers were not significantly different between rVSV-HRTV K1074A and rVSV-HRTV C621S+V753T+K1074A; however, the trend shows higher neutralizing titers on average when mice were vaccinated with rVSV-HRTV K1074A. This suggests that rVSV-HRTV with the single K1074A substitution is superior to the other two mutants tested, which may be a result of the increased surface expression of glycoproteins with this substitution (Fig. 5D) or increased replication *in vivo*. However, it is also important to note that we saw high variability in neutralizing antibody titers in all vaccine groups, which may suggest variable levels of viral replication between immunocompetent individuals. Nevertheless, every animal vaccinated with rVSV-HRTV K1074A developed detectable neutralizing antibodies, which was not the case for animals vaccinated with rVSV-HRTV WT (p3) and rVSV-HRTV C621S+V753T+K1074A (Fig. 6A). These data suggest that rVSV-HRTV K1074A replicates more efficiently *in vivo*.

To directly evaluate replication of the rVSV-HRTV vaccine candidates in animal models, we injected groups of six male and six female AG129 mice with a low dose (10^2^ PFU) of each vaccine candidate via the intraperitoneal route. AG129 mice are deficient in interferon α, β, and γ receptors, which makes them highly susceptible to viral infections and allows observation of viral replication *in vivo* (132, 133). These mice also serve as the established HRTV challenge model (108), allowing evaluation of the vaccine potential for these rVSV-HRTV candidates in a model susceptible to lethal challenge. In response to infection with rVSV-HRTV WT (p3, which carries two spontaneous changes), the mice lost a moderate amount of weight starting at day 3 post-injection, but they all recovered by day 7 (Fig. 6B). This suggests that the virus replicates well initially in these mice before they are able to control replication. Infection with rVSV-HRTV C621S+V753T+K1074A had similar results, but this virus was better tolerated by the mice, and they only lost a small amount of weight (Fig. 6B). In contrast, rVSV-HRTV K1074A immunization resulted in weight loss in the mice similar to that of the rVSV-HRTV WT group (Fig. 6B), but most (10 of the 12 mice) succumbed within 5–7 days post-injection (Fig. 6C). This might suggest that rVSV-HRTV K1074A replicates more efficiently *in vivo* than the other mutants*,* resulting in mortality in severely immunocompromised mice. To directly address *in vivo* replication, we evaluated rVSV viremia in vaccinated mice 2 days post-vaccination (data not shown). We used qPCR to quantify levels of VSV-N RNA in the blood of vaccinated mice relative to the mouse housekeeping gene hypoxanthine guanine phosphoribosyltransferase (mHPRT). The average fold change of VSV-N in vaccinated mice relative to the sham-infected control group was quantified via the 2^−ΔΔCt^ method (116). We found that mice vaccinated with rVSV-HRTV WT or rVSV-HRTV C621S+V753T+K1074A did not exhibit detectable replication on day 2 post-vaccination compared to the control group, with an average fold change of 1.6 or 0.8, respectively. In contrast, we observed significant replication of rVSV-HRTV K1074A 2 days post-vaccination. The average fold change in VSV-N RNA was 103.6 times higher than the sham control group, confirming replication of this rVSV in AG129 mice.

To evaluate the protective efficacy of our rVSV-HRTV candidates, we challenged the AG129 mice that survived rVSV-HRTV vaccination (Fig. 6C) with 10^2^ CCID_50_ of mouse-adapted HRTV (MA-HRTV) via subcutaneous injection (108). It should be noted that the AG129 mouse model has several limitations for vaccine efficacy studies. Their interferon deficiency makes them extremely susceptible to replication of even innocuous viruses, as seen above. The absence of interferon signaling also limits evaluation of T- and B-cell responses since activation of these cells is highly dependent on interferon (134, 135). For this reason, animals deficient in interferon signaling are not ideal for evaluation of vaccine efficacy. Nevertheless, we are limited to this animal model due to the paucity of immunocompetent models for HRTV virus (136). Blood was collected from the surviving vaccinated AG129 mice on day 21 post-vaccination so that neutralizing antibody titers could be correlated with survival (Fig. 6D). All mice vaccinated with rVSV-HRTV WT (p3) developed detectable neutralizing antibodies, and the average neutralizing titer (640) was very high in these mice. Induction of neutralizing antibodies was more variable in the mice vaccinated with rVSV-HRTV K1074A and C621S+V753T+K1074A. Given that the vaccine dose administered was very low (10^2^ PFU), it is possible that these mice did not receive a dose sufficient for immunization, or that some mice were better able to control early replication of the more attenuated rVSV-HRTV C621S+V753T+K1074A. Nevertheless, the average neutralizing titer of mice vaccinated with rVSV-HRTV K1074A was approximately 167, and the neutralizing titer in the C621S+V753T+K1074A group was 481. Mice were challenged on day 23 post-vaccination, and they were evaluated for weight loss (Fig. 6E and F), signs of morbidity, and mortality (Fig. 6G) for 21 days following challenge. As anticipated, the mice vaccinated with the PBS placebo lost weight rapidly starting at day 4 post-challenge and succumbed to viral infection by day 7 post-challenge. All the vaccinated groups had elevated survival rates compared to the PBS placebo. All mice vaccinated with rVSV-HRTV WT (p3) survived challenge with minimal weight loss or morbidity. Only two mice survived vaccination with rVSV-HRTV K1074A (Fig. 6C), and only one of these mice developed neutralizing antibodies (Fig. 6D). That mouse survived the MA-HRTV challenge, but the mouse that did not develop detectable neutralizing antibodies did not survive. The small surviving sample size of rVSV-HRTV K1074A-vaccinated mice limits the conclusions that can be drawn from this group, as the remaining animals are likely not representative of the full group. However, this trend was similar in the rVSV-HRTV C621S+V753T+K1074A group. Seven of the twelve vaccinated mice developed neutralizing antibodies above the limit of detection, and all but one of these mice survived infection. The mouse that seroconverted but succumbed to infection had a neutralizing antibody titer of 40, so it is possible that this low titer was insufficient for protection. Collectively, 20 AG129 mice survived vaccination and developed detectable neutralizing antibodies, and 19 of these survived HRTV challenge. Conversely, all five mice that did not develop neutralizing antibodies succumbed to HRTV infection. Overall, there is a strong positive correlation between the development of neutralizing antibodies and HRTV challenge survival.

As seen in Fig. 6C, the pathogenicity of rVSV-HRTV K1074A in severely immune-deficient AG129 mice makes it difficult to assess the protective efficacy of this vaccine candidate. However, our neutralizing antibody titers from C57BL/6 mice suggest that this vaccine would be the most advantageous in immunocompetent hosts (Fig. 6A). To directly assess whether the antibody titers elicited by rVSV-HRTV K1074A vaccination in C57BL/6 mice would be protective, we performed a passive transfer study in AG129 mice. We used the serum from the day 70 blood collection to compare the efficacy of passive transfer of pooled serum from rVSV-HRTV WT (p3) (pooled neutralizing titer: 25) or rVSV-HRTV K1074A (pooled neutralizing titer: 253) vaccinated C57BL/6 mice. We chose these samples for evaluation as they had the most disparate phenotypes. Four male and four female AG129 mice per group were subcutaneously challenged with 10^2^ CCID_50_ of MA-HRTV on day 0 and were administered a high or low dose of immune serum via IP injection on day 1 post-infection. The high dose consisted of 120 μL undiluted serum, and the low dose consisted of 120 μL (rVSV-HRTV WT) or 110 μL (rVSV-HRTV K1074A) serum diluted by 50%. Additional groups administered 120 μL of non-immune serum or PBS were included as negative controls.

**Fig 7:**
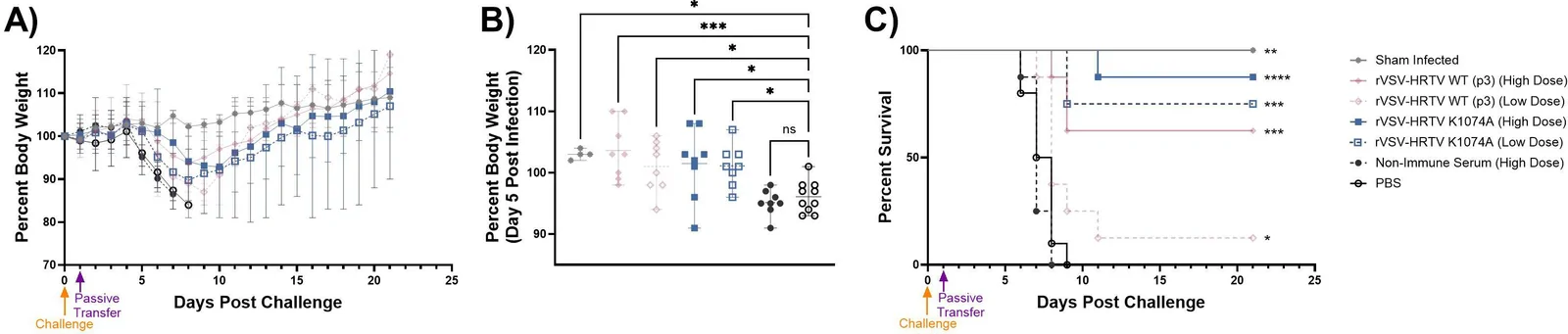
rVSV-HRTV K1074A vaccination results in increased protective efficacy of passive transfer. Four male and four female AG129 mice were subcutaneously challenged with MA-HRTV on day 0 and then IP administered serum from mice vaccinated with rVSV-HRTV WT or K1074A 1 day post-challenge. (**A**) Weight loss (mean and range) is shown for 21 days post-HRTV challenge, and (**B**) compared to the PBS vaccine placebo group at day 5 post-challenge (ordinary one-way ANOVA with Dunnett’s multiple comparisons test; *, *P* < 0.05; ***, *P* < 0.001). (**C**) Survival is shown for 21 days post-HRTV challenge (statistical significance of survival was evaluated via the Mantel-Cox log-rank test compared to mice vaccinated with PBS placebo; *, *P* < 0.05; **, *P* < 0.01; ***, *P* < 0.001; ****, *P* < 0.0001).

As anticipated, the mice administered non-immune serum or PBS were not protected from infection and therefore lost significant weight and succumbed to HRTV infection between 6 and 9 days post-challenge (Fig. 7A through C). Administration of serum from rVSV-HRTV WT (p3) vaccinated mice was partially protective. Five of eight mice given the high dose of rVSV-HRTV WT (p3) serum survived infection despite moderate weight loss. The low dose of rVSV-HRTV WT (p3) serum was substantially less effective, and only one mouse in the group survived infection. As we expected based on the neutralizing titers, serum from rVSV-HRTV K1074A was more efficacious than rVSV-HRTV WT (p3). Seven of eight mice that received the high dose of rVSV-HRTV K1074A serum survived infection, and death was delayed in the mouse that succumbed to infection compared to the placebo groups. The low dose of serum from rVSV-HRTV K1074A-vaccinated mice was also effective, and six mice from this group survived challenge. These data indicate that antibodies administered via passive transfer are sufficient for protection from lethal HRTV challenge.

## DISCUSSION

SFTSV and HRTV remain important emerging tick-borne pathogens for which vaccine development has lagged despite clear public health prioritization (28, 29). A major limitation of rVSV-based vaccine approaches for these viruses has been poor replication and attenuation *in vitro*, particularly for rVSV-SFTSV (63, 64, 107). Although prior studies identified adaptive glycoprotein mutations that arise during passage and improve replication, the basis for these effects and their implications for vaccine design were not well understood. Our findings address this gap by showing that a targeted alteration in the Gc cytoplasmic tail can overcome an important trafficking barrier that limits the vaccine performance of bunyavirus glycoprotein-bearing rVSVs.

A key conclusion of this work is that mutation of a conserved lysine residue in the Gc cytoplasmic tail—K1071A in SFTSV and K1074A in HRTV—is sufficient to improve rVSV performance both *in vitro* and, for HRTV, *in vivo*. In SFTSV, this substitution markedly enhanced pseudotype production and was associated with increased glycoprotein surface expression and incorporation into VSV particles. Mechanistically, our data support a model in which K1071A disrupts a noncanonical COPI-binding signal, reducing interaction with β′-COP and redirecting the Gn/Gc complex from intracellular membranes to the plasma membrane. COPI is a complex of several proteins that functions to recycle cargo from the Golgi to the ER and retain cargo within the ER. Cargo-binding components of COPI typically interact with target proteins bearing di-lysine motifs, either KKxx or KxKxx, that bind to α-COP or β′-COP, respectively. Noncanonical β′-COP targeting sequences, including KxHxx, have been identified in the cytoplasmic tails of other viruses, most notably the betacoronaviruses SARS-CoV, SARS-CoV-2, and MERS (96, 100, 104, 124–126), where production of high-titer VSV pseudotypes required cytoplasmic tail truncation.

Our identification of a COPI retention signal is supported by mutational analysis of the Gc cytoplasmic tail in related bunyaviruses. Mutation of the lysine in the −3 position to alanine (GcK3A) in the Gc cytoplasmic tail of UUKV resulted in surface expression of both Gn and Gc (94). Similarly, alanine substitution of either lysine at the −3 or −5 positions in the RVFV Gc cytoplasmic tail altered localization of Gn and Gc with significant expression at the cell surface (93). Identification of a COPI-based retention signal is significant because it directly links attenuation of bunyavirus glycoprotein-bearing rVSVs to incompatibility between the native trafficking of bunyaviral glycoproteins and the plasma-membrane-budding site of VSV. More broadly, these results suggest that intracellular retention and retrieval signals in glycoproteins from the *Bunyaviracetes* class may represent a general barrier to the development of recombinant VSV vaccines for this virus class as has been hypothesized by us and others (64, 107, 137).

Our rescue and passage experiments further indicate that not all adaptive mutations are equally desirable from a vaccine development standpoint. Although several substitutions improved pseudotype formation, only K1071A in SFTSV and K1074A in HRTV consistently supported recovery of genetically stable recombinant VSV. By contrast, viruses bearing additional substitutions accumulated further mutations during serial passage, suggesting ongoing selective pressure and an unstable genetic background. This distinction is particularly important for vaccine translational development. While spontaneous passage-acquired mutations may transiently improve growth, their instability creates uncertainty regarding manufacturing consistency, biological behavior, and antigenic integrity. In contrast, K1071A/K1074A represent rationally selected modifications with a defined mechanism and reproducible phenotype.

The data also suggest that some of the additional adaptive substitutions, particularly M749T and its HRTV cognate, may improve replication through increased fusogenicity rather than improved compatibility with VSV assembly. Although this may enhance cell-to-cell spread *in vitro*, it is less desirable in a vaccine antigen, as increased triggering of the glycoprotein could favor post-fusion conformations that are less effective at eliciting neutralizing antibodies (138–140). This may explain why adding these mutations did not improve—and in some cases may have reduced—the overall suitability of the recombinant viruses despite their apparent adaptive benefit in culture. While the adaptive mutations aside from K1071A/K1074A that we evaluated (C617R, M749T, and E982K) were not useful for vaccine development, they could be employed for production of high-titer VSVΔG(SFTSV) or VSVΔG(HRTV) pseudotypes, as we observed that a combination of these substitutions improves the recovery of high-titer pseudotypes by nearly 100-fold. This finding is important because VSVΔG(SFTSV) pseudotypes have broad utility as diagnostic tools that allow quantification of neutralizing antibodies or identification of potential antiviral drugs in BSL-2 facilities (141). Overall, our results underscore that improved rVSV replication alone is not sufficient to define an optimal vaccine candidate; the mechanism by which replication is enhanced is also highly relevant.

The HRTV studies strengthen this conclusion by showing that the benefits of the Gc tail substitution extend beyond cell culture. rVSV-HRTV K1074A replicated more efficiently in AG129 mice and elicited stronger neutralizing antibody responses in immunocompetent C57BL/6 mice than the other HRTV constructs tested. Neutralizing antibody titers in all groups were low prior to the booster dose; however, rVSV-HRTV K1074A induced significantly higher titers than either of the other vaccines at day 26, prior to the boost. Poor neutralizing antibody titers following a single rVSV vaccine dose in immunocompetent animals have been previously described for rVSV-SFTSV, and this may be due to attenuated replication of these rVSV vaccines in animals. However, a single dose of rVSV-SFTSV has been shown to be protective in *Ifnar*^−/−^ mice or C57BL/6 mice administered an interferon receptor-blocking antibody (63, 64, 112). Early studies evaluating rVSV-ZEBOV vaccination in mice also demonstrated that neutralizing antibodies were slow to develop, but rapid protection was afforded by rVSV-ZEBOV vaccination nonetheless (142).

Although AG129 mice are not an ideal model for vaccine studies, they are currently the only practical model for lethal HRTV challenge, so findings from this system are still valuable. Mice with impaired interferon signaling are generally suboptimal for vaccine evaluation because interferon responses are important for generating effective antibody and T-cell immunity (134, 135). However, no suitable immunocompetent animal model of HRTV infection is currently available. For example, immunocompetent C57BL/6 mice do not develop detectable viremia or clinical signs of infection, and similar results have been reported in other immunocompetent models (136). HRTV infection has also been studied in STAT2-knockout hamsters, but although these animals develop disease, they do not show substantial mortality, which limits their usefulness for lethal challenge experiments (143). *Ifnar^−/−^* mice, which lack only type I interferon receptors and are therefore less severely immunocompromised than AG129 mice, can be infected only with very high intraperitoneal doses of HRTV, making their use impractical for many studies (144). As a result, AG129 mice remain the only feasible model for lethal challenge studies.

The improved immunogenicity of rVSV-HRTV K1074A in immunocompetent mice, together with the enhanced protection conferred by immune serum from these animals, supports the conclusion that improved *in vivo* replication translated into a more effective humoral response. These findings are especially notable given the absence of any previously reported vaccine directly targeting HRTV infection. Our protection and passive transfer data also provide insight into correlates of protection against HRTV. In the transfer studies, protection tracked broadly with serum neutralizing activity, supporting an important role for antibodies in defense against infection. Similarly, the strong positive correlation of neutralizing antibody development with challenge survival in the AG129 mouse studies underscores the importance of neutralization as an HRTV protection correlate, as is the case for SFTSV (48–53). At the same time, partial protection by passive transfer of serum with low neutralizing titers suggests that non-neutralizing antibody functions may also contribute. This interpretation is consistent with prior work in related systems (145, 146) and suggests that antibody-mediated protection against HRTV may not be fully captured by neutralization assays alone. A more complete understanding of the relative contributions of neutralizing antibodies, Fc-mediated effector functions, and cellular immunity will be important in future studies.

A significant implication of this work is that the strategy described here may be broadly applicable. The lysine-containing motif altered by K1071A/K1074A is conserved across multiple bunyavirus families, raising the possibility that similar trafficking-based modifications could improve glycoprotein display and vector performance for other members of the Bunyaviricetes, including the *Phenuiviridae, Hantaviridae,* and *Peribunyaviridae* families. Such an approach may be relevant not only for rVSV-based vaccines and pseudotype systems but also for other platforms, including mRNA-LNP vaccines, where increased cell-surface expression of bunyaviral glycoproteins could enhance immunogenicity. Future studies should determine how broadly this mechanism applies across bunyaviruses and whether similar modifications can improve vaccine performance without compromising antigenic authenticity.

In summary, our findings argue that intracellular trafficking of bunyaviral glycoproteins is a major and previously underappreciated determinant of rVSV vaccine performance. By identifying K1071A/K1074A as targeted substitutions that improve glycoprotein trafficking, particle incorporation, replication, and genetic stability, this study provides both mechanistic insight and a practical path forward for vaccine optimization. These results establish rVSV-HRTV K1074A as a promising HRTV vaccine candidate and suggest that rational engineering of glycoprotein trafficking signals may represent a broadly useful strategy for advancing vaccines against bunyaviruses.

## Data Availability

All data are provided in the figures of the paper. Additional information is available from the corresponding author upon request.

## SUPPLEMENTAL MATERIAL

Supplemental material jvi.01311-26-s0001.docx Figures S1 and S2.

ASM does not own the copyrights to Supplemental Material that may be linked to, or accessed through, an article. The authors have granted ASM a non-exclusive, world-wide license to publish the Supplemental Material files. Please contact the corresponding author directly for reuse.

## References

[1] Yu X-J, Liang M-F, Zhang S-Y, Liu Y, Li J-D, Sun Y-L, Zhang L, Zhang Q-F, Popov VL, Li C, et al.. 2011. Fever with thrombocytopenia associated with a novel bunyavirus in China. N Engl J Med 364:1523–1532. doi:10.1056/NEJMoa1010095 PMC3113718 21410387

[2] McMullan LK, Folk SM, Kelly AJ, MacNeil A, Goldsmith CS, Metcalfe MG, Batten BC, Albariño CG, Zaki SR, Rollin PE, Nicholson WL, Nichol ST. 2012. A new phlebovirus associated with severe febrile illness in Missouri. N Engl J Med 367:834–841. doi:10.1056/NEJMoa1203378 22931317

[3] Kuhn JH, Brown K, Adkins S, de la Torre JC, Digiaro M, Ergünay K, Firth AE, Hughes HR, Junglen S, Lambert AJ, Maes P, Marklewitz M, Palacios G, Sasaya T, Shi M, Zhang Y-Z, Wolf YI, Turina M. 2024. Promotion of order *Bunyavirales* to class *Bunyaviricetes* to accommodate a rapidly increasing number of related polyploviricotine viruses. J Virol 98:e0106924. doi:10.1128/jvi.01069-24 PMC11494962 39303014

[4] Cui H, Shen S, Chen L, Fan Z, Wen Q, Xing Y, Wang Z, Zhang J, Chen J, La B, Fang Y, Yang Z, Yang S, Yan X, Pei S, Li T, Cui X, Jia Z, Cao W. 2024. Global epidemiology of severe fever with thrombocytopenia syndrome virus in human and animals: a systematic review and meta-analysis. The Lancet Regional Health - Western Pacific 48:101133. doi:10.1016/j.lanwpc.2024.101133 PMC11261768 39040038

[5] Casel MA, Park SJ, Choi YK. 2021. Severe fever with thrombocytopenia syndrome virus: emerging novel phlebovirus and their control strategy. Exp Mol Med 53:713–722. doi:10.1038/s12276-021-00610-1 PMC8178303 33953322

[6] Bopp NE, Kaiser JA, Strother AE, Barrett ADT, Beasley DWC, Benassi V, Milligan GN, Preziosi MP, Reece LM. 2020. Baseline mapping of severe fever with thrombocytopenia syndrome virology, epidemiology and vaccine research and development. NPJ Vaccines 5:111. doi:10.1038/s41541-020-00257-5 PMC7746727 33335100

[7] Takahashi T, Maeda K, Suzuki T, Ishido A, Shigeoka T, Tominaga T, Kamei T, Honda M, Ninomiya D, Sakai T, et al.. 2014. The first identification and retrospective study of severe fever with thrombocytopenia syndrome in Japan. J Infect Dis 209:816–827. doi:10.1093/infdis/jit603 PMC7107388 24231186

[8] Kobayashi Y, Kato H, Yamagishi T, Shimada T, Matsui T, Yoshikawa T, Kurosu T, Shimojima M, Morikawa S, Hasegawa H, Saijo M, Oishi K, Japan S. 2020. Severe fever with thrombocytopenia syndrome, Japan, 2013-2017. Emerg Infect Dis 26:692–699. doi:10.3201/eid2604.191011 PMC7101122 32186502

[9] Kim K-H, Yi J, Kim G, Choi SJ, Jun KI, Kim N-H, Choe PG, Kim N-J, Lee J-K, Oh M. 2013. Severe fever with thrombocytopenia syndrome, South Korea, 2012. Emerg Infect Dis 19:1892–1894. doi:10.3201/eid1911.130792 PMC3837670 24206586

[10] Park SW, Han MG, Yun SM, Park C, Lee WJ, Ryou J. 2014. Severe fever with thrombocytopenia syndrome virus, South Korea, 2013. Emerg Infect Dis 20:1880–1882. doi:10.3201/eid2011.140888 PMC4214315 25341085

[11] Tran XC, Yun Y, Van An L, Kim S-H, Thao NTP, Man PKC, Yoo JR, Heo ST, Cho N-H, Lee KH. 2019. Endemic severe fever with thrombocytopenia syndrome, Vietnam. Emerg Infect Dis 25:1029–1031. doi:10.3201/eid2505.181463 PMC6478219 31002059

[12] Peng SH, Yang SL, Tang SE, Wang TC, Hsu TC, Su CL, Chen MY, Shimojima M, Yoshikawa T, Shu PY. 2020. Human case of severe fever with thrombocytopenia syndrome virus infection, Taiwan, 2019. Emerg Infect Dis 26:1612–1614. doi:10.3201/eid2607.200104 PMC7323535 32568054

[13] Zohaib A, Zhang J, Saqib M, Athar MA, Hussain MH, Chen J, Sial A-U-R, Tayyab MH, Batool M, Khan S, et al.. 2020. Serologic evidence of severe fever with thrombocytopenia syndrome virus and related viruses in Pakistan. Emerg Infect Dis 26:1513–1516. doi:10.3201/eid2607.190611 PMC7323538 32568060

[14] Win AM, Nguyen YTH, Kim Y, Ha NY, Kang JG, Kim H, San B, Kyaw O, Htike WW, Choi DO, Lee KH, Cho NH. 2020. Genotypic heterogeneity of *Orientia tsutsugamushi* in scrub typhus patients and thrombocytopenia syndrome co-infection, Myanmar. Emerg Infect Dis 26:1878–1881. doi:10.3201/eid2608.200135 PMC7392420 32687023

[15] Saba Villarroel PM, Chaiphongpachara T, Nurtop E, Laojun S, Pangpoo-Nga T, Songhong T, Supungul D, Baronti C, Thirion L, Leaungwutiwong P, de Lamballerie X, Missé D, Wichit S. 2024. Seroprevalence study in humans and molecular detection in *Rhipicephalus sanguineus* ticks of severe fever with thrombocytopenia syndrome virus in Thailand. Sci Rep 14:13397. doi:10.1038/s41598-024-64242-x PMC11167008 38862576

[16] Zheng W, Zhong F, Wang W, Liu H, Peng Y, Carr MJ, Peng Z, Shi W. 2026. SFTS exerts an underrecognized disease burden and socioeconomic effect in East Asia. Biosaf Health 8:15–19. doi:10.1016/j.bsheal.2025.12.006 PMC12931377 41743029

[17] Sun J, Lu L, Wu H, Yang J, Ren J, Liu Q. 2017. The changing epidemiological characteristics of severe fever with thrombocytopenia syndrome in China, 2011–2016. Sci Rep 7:9236. doi:10.1038/s41598-017-08042-6 PMC5569157 28835633

[18] Yamanaka A, Kirino Y, Fujimoto S, Ueda N, Himeji D, Miura M, Sudaryatma PE, Sato Y, Tanaka H, Mekata H, Okabayashi T. 2020. Direct transmission of severe fever with thrombocytopenia syndrome virus from domestic cat to veterinary personnel. Emerg Infect Dis 26:2994–2998. doi:10.3201/eid2612.191513 PMC7706950 33219655

[19] Tsuru M, Suzuki T, Murakami T, Matsui K, Maeda Y, Yoshikawa T, Kurosu T, Shimojima M, Shimada T, Hasegawa H, Maeda K, Morikawa S, Saijo M. 2021. Pathological characteristics of a patient with severe fever with thrombocytopenia syndrome (SFTS) infected with SFTS virus through a sick cat’s bite. Viruses 13:204. doi:10.3390/v13020204 PMC7912689 33572914

[20] Kirino Y, Ishijima K, Miura M, Nomachi T, Mazimpaka E, Sudaryatma PE, Yamanaka A, Maeda K, Sugimoto T, Saito A, Mekata H, Okabayashi T. 2021. Seroprevalence of severe fever with thrombocytopenia syndrome virus in small-animal veterinarians and nurses in the Japanese prefecture with the highest case load. Viruses 13:229. doi:10.3390/v13020229 PMC7912989 33540629

[21] Jiang XL, Zhang S, Jiang M, Bi ZQ, Liang MF, Ding SJ, Wang SW, Liu JY, Zhou SQ, Zhang XM, Li DX, Xu AQ. 2015. A cluster of person-to-person transmission cases caused by SFTS virus in Penglai, China. Clin Microbiol Infect 21:274–279. doi:10.1016/j.cmi.2014.10.006 25687766

[22] Jung IY, Choi W, Kim J, Wang E, Park SW, Lee WJ, Choi JY, Kim HY, Uh Y, Kim YK. 2019. Nosocomial person-to-person transmission of severe fever with thrombocytopenia syndrome. Clin Microbiol Infect 25:633. doi:10.1016/j.cmi.2019.01.006 30677496

[23] Kim WY, Choi W, Park SW, Wang EB, Lee WJ, Jee Y, Lim KS, Lee HJ, Kim SM, Lee SO, Choi SH, Kim YS, Woo JH, Kim SH. 2015. Nosocomial transmission of severe fever with thrombocytopenia syndrome in Korea. Clin Infect Dis 60:1681–1683. doi:10.1093/cid/civ128 25694652

[24] Wen Y, Fang Y, Cao F, Zhang G, Cheng S, Yu Y, Huang R, Ni Z, Li J. 2024. A person-to-person transmission cluster of severe fever with thrombocytopenia syndrome characterized by mixed viral infections with familial and nosocomial clustering. Heliyon 10:e24502. doi:10.1016/j.heliyon.2024.e24502 PMC10827760 38298613

[25] Zheng W, Zhou H, Carr MJ, Shi W. 2026. Epidemic potential of SFTSV: increasing evidence for non-vector-borne transmission. Lancet Microbe 7:101271. doi:10.1016/j.lanmic.2025.101271 41205620

[26] Luo L-M, Zhao L, Wen H-L, Zhang Z-T, Liu J-W, Fang L-Z, Xue Z-F, Ma D-Q, Zhang X-S, Ding S-J, Lei X-Y, Yu X. 2015. Haemaphysalis longicornis ticks as reservoir and vector of severe fever with thrombocytopenia syndrome virus in China. Emerg Infect Dis 21:1770–1776. doi:10.3201/eid2110.150126 PMC4593435 26402039

[27] Li JC, Zhao J, Li H, Fang LQ, Liu W. 2022. Epidemiology, clinical characteristics, and treatment of severe fever with thrombocytopenia syndrome. Infectious Medicine 1:40–49. doi:10.1016/j.imj.2021.10.001 PMC10699716 38074982

[28] Mehand MS, Al-Shorbaji F, Millett P, Murgue B. 2018. The WHO R&D blueprint: 2018 review of emerging infectious diseases requiring urgent research and development efforts. Antiviral Res 159:63–67. doi:10.1016/j.antiviral.2018.09.009 PMC7113760 30261226

[29] NIAID. 2024. NIAID biodefense pathogens. Available from: https://www.niaid.nih.gov/research/niaid-biodefense-pathogens. Retrieved 7 Jan 2025.

[30] CDC. 2024. Heartland virus - data and maps for heartland, on US centers for disease control and prevention. Available from: https://www.cdc.gov/heartland-virus/data-maps/index.html. Retrieved 11 Jun 2025.

[31] Mantlo EK, Haley NJ. 2023. Heartland virus: an evolving story of an emerging zoonotic and vector-borne disease. Zoonotic Diseases 3:188–202. doi:10.3390/zoonoticdis3030016

[32] Dembek ZF, Mothershead JL, Cirimotich CM, Wu A. 2024. Heartland virus disease-an underreported emerging infection. Microorganisms 12:286. doi:10.3390/microorganisms12020286 PMC10892980 38399689

[33] Lindsey NP, Menitove JE, Biggerstaff BJ, Turabelidze G, Parton P, Peck K, Basile AJ, Kosoy OI, Fischer M, Staples JE. 2019. Seroprevalence of Heartland virus antibodies in blood donors, Northwestern Missouri, USA. Emerg Infect Dis 25:358–360. doi:10.3201/eid2502.181288 PMC6346440 30511916

[34] Bosco-Lauth AM, Panella NA, Root JJ, Gidlewski T, Lash RR, Harmon JR, Burkhalter KL, Godsey MS, Savage HM, Nicholson WL, Komar N, Brault AC. 2015. Serological investigation of heartland virus *(Bunyaviridae*: Phlebovirus) exposure in wild and domestic animals adjacent to human case sites in Missouri 2012–2013. Am J Trop Med Hyg 92:1163–1167. doi:10.4269/ajtmh.14-0702 PMC4458820 25870419

[35] Clarke LL, Ruder MG, Mead DG, Howerth EW. 2018. Heartland virus exposure in white-tailed deer in the Southeastern United States, 2001–2015. Am J Trop Med Hyg 99:1346–1349. doi:10.4269/ajtmh.18-0555 PMC6221220 30255829

[36] Riemersma KK, Komar N. 2015. Heartland virus neutralizing antibodies in vertebrate wildlife, United States, 2009-2014. Emerg Infect Dis 21:1830–1833. doi:10.3201/eid2110.150380 PMC4593439 26401988

[37] Kim D, Lai CJ, Cha I, Jung JU. 2024. Current progress of severe fever with thrombocytopenia syndrome virus (SFTSV) vaccine development. Viruses 16:128. doi:10.3390/v16010128 PMC10818334 38257828

[38] Elliott RM, Schmaljohn CS. 2013. Bunyaviridae. In Knipe DM, Howley PM (ed), Fields Virology, Sixth edition. Vol. 1. Wolters Kluwer Health/Lippincott Williams & Wilkins, Philadelphia, PA.

[39] Lozach P-Y, Kühbacher A, Meier R, Mancini R, Bitto D, Bouloy M, Helenius A. 2011. DC-SIGN as a receptor for phleboviruses. Cell Host Microbe 10:75–88. doi:10.1016/j.chom.2011.06.007 21767814

[40] Hofmann H, Li X, Zhang X, Liu W, Kühl A, Kaup F, Soldan SS, González-Scarano F, Weber F, He Y, Pöhlmann S. 2013. Severe fever with thrombocytopenia virus glycoproteins are targeted by neutralizing antibodies and can use DC-SIGN as a receptor for pH-dependent entry into human and animal cell lines. J Virol 87:4384–4394. doi:10.1128/JVI.02628-12 PMC3624395 23388721

[41] Halldorsson S, Behrens A-J, Harlos K, Huiskonen JT, Elliott RM, Crispin M, Brennan B, Bowden TA. 2016. Structure of a phleboviral envelope glycoprotein reveals a consolidated model of membrane fusion. Proc Natl Acad Sci USA 113:7154–7159. doi:10.1073/pnas.1603827113 PMC4932967 27325770

[42] Zhang L, Peng X, Wang Q, Li J, Lv S, Han S, Zhang L, Ding H, Wang CY, Xiao G, Du X, Peng K, Li H, Liu W. 2023. CCR2 is a host entry receptor for severe fever with thrombocytopenia syndrome virus. Sci Adv 9:eadg6856. doi:10.1126/sciadv.adg6856 PMC10396298 37531422

[43] Zhu Y, Wu Y, Chai Y, Qi J, Peng R, Feng WH, Gao GF. 2018. The postfusion structure of the heartland virus Gc glycoprotein supports taxonomic separation of the bunyaviral families *Phenuiviridae* and *Hantaviridae*. J Virol 92. doi:10.1128/JVI.01558-17 PMC5730780 29070692

[44] Drake MJ, Brennan B, Briley K, Bart SM, Sherman E, Szemiel AM, Minutillo M, Bushman FD, Bates P. 2017. A role for glycolipid biosynthesis in severe fever with thrombocytopenia syndrome virus entry. Pathog 13:e1006316. doi:10.1371/journal.ppat.1006316 PMC5397019 28388693

[45] Sun Y, Qi Y, Liu C, Gao W, Chen P, Fu L, Peng B, Wang H, Jing Z, Zhong G, Li W. 2014. Nonmuscle myosin heavy chain IIA is a critical factor contributing to the efficiency of early infection of severe fever with thrombocytopenia syndrome virus. J Virol 88:237–248. doi:10.1128/JVI.02141-13 PMC3911693 24155382

[46] Xia T, Wu X, Hong E, Jung K, Lai CJ, Kwak MJ, Seo H, Kim S, Jiang Z, Cha I, Jung JU. 2023. Glucosylceramide is essential for Heartland and Dabie bandavirus glycoprotein-induced membrane fusion. Pathog 19:e1011232. doi:10.1371/journal.ppat.1011232 PMC10016662 36920967

[47] Kimura M, Egawa K, Ozawa T, Kishi H, Shimojima M, Taniguchi S, Fukushi S, Fujii H, Yamada H, Tan L, Sano K, Katano H, Suzuki T, Morikawa S, Saijo M, Tani H. 2021. Characterization of pseudotyped vesicular stomatitis virus bearing the Heartland virus envelope glycoprotein. Virology 556:124–132. doi:10.1016/j.virol.2020.10.006 33561699

[48] Wu X, Li Y, Huang B, Ma X, Zhu L, Zheng N, Xu S, Nawaz W, Xu C, Wu Z. 2020. A single-domain antibody inhibits SFTSV and mitigates virus-induced pathogenesis *in vivo*. JCI Insight 5. doi:10.1172/jci.insight.136855 PMC7406269 32641581

[49] Wu Y, Zhu Y, Gao F, Jiao Y, Oladejo BO, Chai Y, Bi Y, Lu S, Dong M, Zhang C, Huang G, Wong G, Li N, Zhang Y, Li Y, Feng W, Shi Y, Liang M, Zhang R, Qi J, Gao GF. 2017. Structures of phlebovirus glycoprotein Gn and identification of a neutralizing antibody epitope. Proc Natl Acad Sci USA 114:E7564–E7573. doi:10.1073/pnas.1705176114 PMC5594662 28827346

[50] Kim KH, Kim J, Ko M, Chun JY, Kim H, Kim S, Min J-Y, Park WB, Oh M, Chung J. 2019. An anti-Gn glycoprotein antibody from a convalescent patient potently inhibits the infection of severe fever with thrombocytopenia syndrome virus. PLoS Pathog 15:e1007375. doi:10.1371/journal.ppat.1007375 PMC6380599 30707748

[51] Guo X, Zhang L, Zhang W, Chi Y, Zeng X, Li X, Qi X, Jin Q, Zhang X, Huang M, Wang H, Chen Y, Bao C, Hu J, Liang S, Bao L, Wu T, Zhou M, Jiao Y. 2013. Human antibody neutralizes severe fever with thrombocytopenia syndrome virus, an emerging hemorrhagic fever virus. Clin Vaccine Immunol 20:1426–1432. doi:10.1128/CVI.00222-13 PMC3889583 23863504

[52] Shimojima M, Sugimoto S, Umekita K, Onodera T, Sano K, Tani H, Takamatsu Y, Yoshikawa T, Kurosu T, Suzuki T, Takahashi Y, Ebihara H, Saijo M. 2022. Neutralizing mAbs against SFTS virus Gn protein show strong therapeutic effects in an SFTS animal model. Viruses 14:1665. doi:10.3390/v14081665 PMC9416629 36016286

[53] Song P, Zheng N, Liu Y, Tian C, Wu X, Ma X, Chen D, Zou X, Wang G, Wang H, Zhang Y, Lu S, Wu C, Wu Z. 2018. Deficient humoral responses and disrupted B-cell immunity are associated with fatal SFTSV infection. Nat Commun 9:3328. doi:10.1038/s41467-018-05746-9 PMC6102208 30127439

[54] Kwak JE, Kim YI, Park SJ, Yu MA, Kwon HI, Eo S, Kim TS, Seok J, Choi WS, Jeong JH, Lee H, Cho Y, Kwon JA, Jeong M, Maslow JN, Kim YE, Jeon H, Kim KK, Shin EC, Song MS, Jung JU, Choi YK, Park SH. 2019. Development of a SFTSV DNA vaccine that confers complete protection against lethal infection in ferrets. Nat Commun 10:3836. doi:10.1038/s41467-019-11815-4 PMC6707330 31444366

[55] Kang JG, Jeon K, Choi H, Kim Y, Kim HI, Ro HJ, Seo YB, Shin J, Chung J, Jeon YK, Kim YS, Lee KH, Cho NH. 2020. Vaccination with single plasmid DNA encoding IL-12 and antigens of severe fever with thrombocytopenia syndrome virus elicits complete protection in IFNAR knockout mice. Negl Trop Dis 14:e0007813. doi:10.1371/journal.pntd.0007813 PMC7112229 32196487

[56] Kim D, Lai CJ, Cha I, Kang S, Yang WS, Choi Y, Jung JU. 2023. SFTSV Gn‐Head mRNA vaccine confers efficient protection against lethal viral challenge. J Med Virol 95:e29203. doi:10.1002/jmv.29203 37909776

[5] Lu J, Liu J, Wu Y, He X, Gao X, Chen X, Chen S, Zhu X, Peng Y, Xiao G, Pan X. 2024. A full-length glycoprotein mRNA vaccine confers complete protection against severe fever with thrombocytopenia syndrome virus, with broad-spectrum protective effects against bandaviruses. J Virol 98. doi:10.1128/jvi.00769-24 PMC11265342 38829138

[58] Kim JY, Jeon K, Park SI, Bang YJ, Park HJ, Kwak HW, Kim DH, Lee SY, Choi EJ, Cho NH, Nam JH. 2023. mRNA vaccine encoding Gn provides protection against severe fever with thrombocytopenia syndrome virus in mice. NPJ Vaccines 8:167. doi:10.1038/s41541-023-00771-2 PMC10618158 37907507

[59] Liu R, Huang DD, Bai JY, Zhuang L, Lu QB, Zhang XA, Liu W, Wang JY, Cao WC. 2015. Immunization with recombinant SFTSV/NSs protein does not promote virus clearance in SFTSV-infected C57BL/6J mice. Viral Immunol 28:113–122. doi:10.1089/vim.2014.0100 PMC4334103 25594805

[60] Kim D, Kim E, Kim S, Chung Y, Lai CJ, Cha I, Cho SD, Choi Y, Dai X, Kim S, Kang S, Kwak MJ, Liu Z, Choi Y, Park SH, Choi YK, Jung JU. 2023. Self-assembling Gn head ferritin nanoparticle vaccine provides full protection from lethal challenge of *Dabie bandavirus* in aged ferrets. mBio 14:e0186823. doi:10.1128/mbio.01868-23 PMC10653821 37712692

[61] Li A, Dai X, Chen L, Liu L, Li C, Liu Y, Wu W, Huang X, Li J, Wang S, Liang M, Li X, Li D. 2022. Immunogenicity and protective efficacy of an inactivated SFTS vaccine candidate in mice. Biosafety and Health 4:45–52. doi:10.1016/j.bsheal.2021.12.008

[62] Yu KM, Park SJ, Yu MA, Kim YI, Choi Y, Jung JU, Brennan B, Choi YK. 2019. Cross-genotype protection of live-attenuated vaccine candidate for severe fever with thrombocytopenia syndrome virus in a ferret model. Proc Natl Acad Sci USA 116:26900–26908. doi:10.1073/pnas.1914704116 PMC6936527 31818942

[63] Dong F, Li D, Wen D, Li S, Zhao C, Qi Y, Jangra RK, Wu C, Xia D, Zhang X, Deng F, Chandran K, Zou Z, Yuan F, Zheng A. 2019. Single dose of a rVSV-based vaccine elicits complete protection against severe fever with thrombocytopenia syndrome virus. NPJ Vaccines 4:5. doi:10.1038/s41541-018-0096-y PMC6347601 30701094

[64] Hicks P, Manzoni TB, Westover JB, Petch RJ, Roper B, Gowen BB, Bates P. 2024. Safety, immunogenicity, and efficacy of a recombinant vesicular stomatitis virus vectored vaccine against severe fever with thrombocytopenia syndrome virus and Heartland bandavirus. Vaccines (Basel) 12:1403. doi:10.3390/vaccines12121403 PMC11728676 39772063

[65] Tian L, Yan L, Zheng W, Lei X, Fu Q, Xue X, Wang X, Xia X, Zheng X. 2021. A rabies virus vectored severe fever with thrombocytopenia syndrome (SFTS) bivalent candidate vaccine confers protective immune responses in mice. Vet Microbiol 257:109076. doi:10.1016/j.vetmic.2021.109076 33957572

[66] Kim J-Y, Jeon K, Hong JJ, Park S-I, Cho H, Park H-J, Kwak HW, Park H-J, Bang Y-J, Lee Y-S, et al.. 2023. Heterologous vaccination utilizing viral vector and protein platforms confers complete protection against SFTSV. Sci Rep 13:8189. doi:10.1038/s41598-023-35328-9 PMC10199661 37210393

[67] Yoshikawa T, Taniguchi S, Kato H, Iwata-Yoshikawa N, Tani H, Kurosu T, Fujii H, Omura N, Shibamura M, Watanabe S, Egawa K, Inagaki T, Sugimoto S, Phanthanawiboon S, Harada S, Yamada S, Fukushi S, Morikawa S, Nagata N, Shimojima M, Saijo M. 2021. A highly attenuated vaccinia virus strain LC16m8-based vaccine for severe fever with thrombocytopenia syndrome. PLoS Pathog 17:e1008859. doi:10.1371/journal.ppat.1008859 PMC7886154 33534867

[68] Kisakov DN, Belyakov IM, Kisakova LA, Yakovlev VA, Tigeeva EV, Karpenko LI. 2024. The use of electroporation to deliver DNA-based vaccines. Expert Rev Vaccines 23:102–123. doi:10.1080/14760584.2023.2292772 38063059

[69] Huang X, Li J, Li A, Wang S, Li D. 2021. Epidemiological characteristics of severe fever with thrombocytopenia syndrome from 2010 to 2019 in mainland China. Int J Environ Res Public Health 18:3092. doi:10.3390/ijerph18063092 PMC8002760 33802869

[70] Roberts A, Buonocore L, Price R, Forman J, Rose JK. 1999. Attenuated vesicular stomatitis viruses as vaccine vectors. J Virol 73:3723–3732. doi:10.1128/JVI.73.5.3723-3732.1999 PMC104148 10196265

[71] Henao-Restrepo AM, Camacho A, Longini IM, Watson CH, Edmunds WJ, Egger M, Carroll MW, Dean NE, Diatta I, Doumbia M, et al.. 2017. Efficacy and effectiveness of an rVSV-vectored vaccine in preventing Ebola virus disease: final results from the Guinea ring vaccination, open-label, cluster-randomised trial (Ebola Ça Suffit!). Lancet 389:505–518. doi:10.1016/S0140-6736(16)32621-6 PMC5364328 28017403

[72] Meakin S, Nsio J, Camacho A, Kitenge R, Coulborn RM, Gignoux E, Johnson J, Sterk E, Musenga EM, Mustafa SHB, Finger F, Ahuka-Mundeke S. 2024. Effectiveness of rVSV-ZEBOV vaccination during the 2018–20 Ebola virus disease epidemic in the Democratic Republic of the Congo: a retrospective test-negative study. Lancet Infect Dis 24:1357–1365. doi:10.1016/S1473-3099(24)00419-5 39178866

[73] Huttner A, Agnandji ST, Engler O, Hooper JW, Kwilas S, Ricks K, Clements TL, Jonsdottir HR, Nakka SS, Rothenberger S, et al.. 2023. Antibody responses to recombinant vesicular stomatitis virus-Zaire Ebolavirus vaccination for Ebola virus disease across doses and continents: 5-year durability. Clinical Microbiology and Infection 29:1587–1594. doi:10.1016/j.cmi.2023.08.026 37661067

[74] Wiedemann A, Lhomme E, Huchon M, Foucat E, Bérerd-Camara M, Guillaumat L, Yaradouno M, Tambalou J, Rodrigues C, Ribeiro A, Béavogui AH, Lacabaratz C, Thiébaut R, Richert L, Lévy Y, Prevac study team. 2024. Long-term cellular immunity of vaccines for Zaire ebola virus diseases. Nat Commun 15:7666. doi:10.1038/s41467-024-51453-z PMC11372064 39227399

[75] Feldmann H, Jones SM, Daddario-DiCaprio KM, Geisbert JB, Ströher U, Grolla A, Bray M, Fritz EA, Fernando L, Feldmann F, Hensley LE, Geisbert TW. 2007. Effective post-exposure treatment of Ebola infection. PLoS Pathog 3:e2. doi:10.1371/journal.ppat.0030002 PMC1779298 17238284

[76] Marzi A, Hanley PW, Haddock E, Martellaro C, Kobinger G, Feldmann H. 2016. Efficacy of vesicular stomatitis virus–ebola virus postexposure treatment in Rhesus macaques infected with Ebola virus Makona. J Infect Dis 214:S360–S366. doi:10.1093/infdis/jiw218 PMC5050468 27496978

[77] Marzi Andrea, Jankeel A, Menicucci AR, Callison J, O’Donnell KL, Feldmann F, Pinski AN, Hanley PW, Messaoudi I. 2021. Single dose of a VSV-based vaccine rapidly protects macaques from Marburg virus disease. Front Immunol 12:774026. doi:10.3389/fimmu.2021.774026 PMC8578864 34777392

[78] Günther S, Feldmann H, Geisbert TW, Hensley LE, Rollin PE, Nichol ST, Ströher U, Artsob H, Peters CJ, Ksiazek TG, Becker S, ter Meulen J, Ölschläger S, Schmidt-Chanasit J, Sudeck H, Burchard GD, Schmiedel S. 2011. Management of accidental exposure to ebola virus in the biosafety level 4 laboratory, Hamburg, Germany. J Infect Dis 204:S785–S790. doi:10.1093/infdis/jir298 21987751

[79] Lai L, Davey R, Beck A, Xu Y, Suffredini AF, Palmore T, Kabbani S, Rogers S, Kobinger G, Alimonti J, Link CJ Jr, Rubinson L, Ströher U, Wolcott M, Dorman W, Uyeki TM, Feldmann H, Lane HC, Mulligan MJ. 2015. Emergency postexposure vaccination with vesicular stomatitis virus-vectored Ebola vaccine after needlestick. JAMA 313:1249–1255. doi:10.1001/jama.2015.1995 PMC4874522 25742465

[80] Wong KK, Davey RT Jr, Hewlett AL, Kraft CS, Mehta AK, Mulligan MJ, Beck A, Dorman W, Kratochvil CJ, Lai L, Palmore TN, Rogers S, Smith PW, Suffredini AF, Wolcott M, Ströher U, Uyeki TM. 2016. Use of postexposure prophylaxis after occupational exposure to Zaire ebolavirus. Clin Infect Dis 63:376–379. doi:10.1093/cid/ciw256 PMC4946014 27118786

[81] Davis C, Tipton T, Sabir S, Aitken C, Bennett S, Becker S, Evans T, Fehling SK, Gunson R, Hall Y, Jackson C, Johanssen I, Kieny MP, Mcmenamin J, Spence E, Strecker T, Sykes C, Templeton K, Thorburn F, Peters E, Henao Restrepo AM, White B, Zambon M, Carroll MW, Thomson EC. 2020. Postexposure prophylaxis with rVSV-ZEBOV following exposure to a patient with Ebola virus disease relapse in the United Kingdom: an operational, safety, and immunogenicity report. Clin Infect Dis 71:2872–2879. doi:10.1093/cid/ciz1165 PMC7778350 31784751

[82] Alabi A, Kokou K, Mahmoudou S, Kavishna R, Nakka SS, Rothenberger S, Musangomunei FP, Olubiyi BF, Bie-Ondo JC, Kabwende AL, Velavan TP, Medaglini D, Nakaya HI, Engler O, Harandi AM, Siegrist C-A, Kremsner PG, Agnandji ST, VSV-EBOPLUS Consortium. 2024. Replication, safety and immunogenicity of the vectored Ebola vaccine rVSV-ΔG-ZEBOV-GP in a sub-Saharan African paediatric population: a randomised controlled, open-label trial in children aged 1-12 years living in Lambaréné, Gabon. J Infect 89:106237. doi:10.1016/j.jinf.2024.106237 39121969

[83] Navaratnarajah CK, Warrier R, Kuhn RJ. 2008. Assembly of viruses: enveloped particles, p 193–200. In Mahy Brian WJ, Van Regenmortel Marc HV (ed), Encyclopedia of Virology, Third Edition. Academic Press.

[84] Plegge T, Hofmann-Winkler H, Spiegel M, Pöhlmann S. 2016. Evidence that processing of the severe fever with thrombocytopenia syndrome virus Gn/Gc polyprotein is critical for viral infectivity and requires an internal Gc signal peptide. PLoS One 11:e0166013. doi:10.1371/journal.pone.0166013 PMC5113920 27855227

[85] Pensiero MN, Hay J. 1992. The Hantaan virus M-segment glycoproteins G1 and G2 can be expressed independently. J Virol 66:1907–1914. doi:10.1128/jvi.66.4.1907-1914.1992 PMC288978 1548747

[86] Haferkamp S, Fernando L, Schwarz TF, Feldmann H, Flick R. 2005. Intracellular localization of Crimean-Congo Hemorrhagic Fever (CCHF) virus glycoproteins. Virol J 2:42. doi:10.1186/1743-422X-2-42 PMC1090624 15850490

[87] Bupp K, Stillmock K, González-Scarano F. 1996. Analysis of the intracellular transport properties of recombinant La Crosse virus glycoproteins. Virology 220:485–490. doi:10.1006/viro.1996.0336 8661399

[88] Gerrard SR, Nichol ST. 2002. Characterization of the Golgi retention motif of Rift Valley fever virus G(N) glycoprotein. J Virol 76:12200–12210. doi:10.1128/jvi.76.23.12200-12210.2002 PMC136907 12414959

[89] Lappin DF, Nakitare GW, Palfreyman JW, Elliott RM. 1994. Localization of Bunyamwera bunyavirus G1 glycoprotein to the Golgi requires association with G2 but not with NSm. J Gen Virol 75 (Pt 12):3441–3451. doi:10.1099/0022-1317-75-12-3441 7996137

[90] Melin L, Persson R, Andersson A, Bergström A, Rönnholm R, Pettersson RF. 1995. The membrane glycoprotein G1 of Uukuniemi virus contains a signal for localization to the Golgi complex. Virus Res 36:49–66. doi:10.1016/0168-1702(95)00006-c PMC7133855 7625126

[91] Matsuoka Y, Chen SY, Compans RW. 1994. A signal for Golgi retention in the bunyavirus G1 glycoprotein. J Biol Chem 269:22565–22573. doi:10.1016/S0021-9258(17)31683-6 8077205

[92] Shi X, Lappin DF, Elliott RM. 2004. Mapping the golgi targeting and retention signal of bunyamwera virus glycoproteins. J Virol 78:10793–10802. doi:10.1128/JVI.78.19.10793-10802.2004 PMC516397 15367646

[93] Carnec X, Ermonval M, Kreher F, Flamand M, Bouloy M. 2014. Role of the cytosolic tails of Rift Valley fever virus envelope glycoproteins in viral morphogenesis. Virology 448:1–14. doi:10.1016/j.virol.2013.09.023 24314631

[94] Overby AK, Popov VL, Pettersson RF, Neve EPA. 2007. The cytoplasmic tails of Uukuniemi Virus (*Bunyaviridae*) G(N) and G(C) glycoproteins are important for intracellular targeting and the budding of virus-like particles. J Virol 81:11381–11391. doi:10.1128/JVI.00767-07 PMC2045573 17670814

[95] Spiegel M, Plegge T, Pöhlmann S. 2016. The role of phlebovirus glycoproteins in viral entry, assembly and release. Viruses 8:202. doi:10.3390/v8070202 PMC4974537 27455305

[96] Jackson LP, Lewis M, Kent HM, Edeling MA, Evans PR, Duden R, Owen DJ. 2012. Molecular basis for recognition of dilysine trafficking motifs by COPI. Dev Cell 23:1255–1262. doi:10.1016/j.devcel.2012.10.017 PMC3521961 23177648

[97] Ma W, Goldberg J. 2013. Rules for the recognition of dilysine retrieval motifs by coatomer. EMBO J 32:926–937. doi:10.1038/emboj.2013.41 PMC3616288 23481256

[98] Nilsson T, Jackson M, Peterson PA. 1989. Short cytoplasmic sequences serve as retention signals for transmembrane proteins in the endoplasmic reticulum. Cell 58:707–718. doi:10.1016/0092-8674(89)90105-0 2527615

[99] Barlowe CK, Miller EA. 2013. Secretory protein biogenesis and traffic in the early secretory pathway. Genetics 193:383–410. doi:10.1534/genetics.112.142810 PMC3567731 23396477

[100] Cattin-Ortolá J, Welch LG, Maslen SL, Papa G, James LC, Munro S. 2021. Sequences in the cytoplasmic tail of SARS-CoV-2 Spike facilitate expression at the cell surface and syncytia formation. Nat Commun 12:5333. doi:10.1038/s41467-021-25589-1 PMC8429659 34504087

[101] Dey D, Singh S, Khan S, Martin M, Schnicker NJ, Gakhar L, Pierce BG, Hasan SS. 2022. An extended motif in the SARS-CoV-2 spike modulates binding and release of host coatomer in retrograde trafficking. Commun Biol 5:115. doi:10.1038/s42003-022-03063-y PMC8825798 35136165

[102] Jennings BC, Kornfeld S, Doray B. 2021. A weak COPI binding motif in the cytoplasmic tail of SARS-CoV-2 spike glycoprotein is necessary for its cleavage, glycosylation, and localization. FEBS Lett 595:1758–1767. doi:10.1002/1873-3468.14109 PMC8209879 33991349

[103] Lontok E, Corse E, Machamer CE. 2004. Intracellular targeting signals contribute to localization of coronavirus spike proteins near the virus assembly site. J Virol 78:5913–5922. doi:10.1128/JVI.78.11.5913-5922.2004 PMC415842 15140989

[104] McBride CE, Li J, Machamer CE. 2007. The cytoplasmic tail of the severe acute respiratory syndrome coronavirus spike protein contains a novel endoplasmic reticulum retrieval signal that binds COPI and promotes interaction with membrane protein. J Virol 81:2418–2428. doi:10.1128/JVI.02146-06 PMC1865919 17166901

[105] Schwegmann-Wessels C, Al-Falah M, Escors D, Wang Z, Zimmer G, Deng H, Enjuanes L, Naim HY, Herrler G. 2004. A novel sorting signal for intracellular localization is present in the S protein of a porcine coronavirus but absent from severe acute respiratory syndrome-associated coronavirus. J Biol Chem 279:43661–43666. doi:10.1074/jbc.M407233200 PMC8060824 15304515

[106] Winter C, Schwegmann-Wessels C, Neumann U, Herrler G. 2008. The spike protein of infectious bronchitis virus is retained intracellularly by a tyrosine motif. J Virol 82:2765–2771. doi:10.1128/JVI.02064-07 PMC2258963 18094153

[107] Hu Q, Zhang Y, Jiang J, Zheng A. 2023. Two point mutations in the glycoprotein of SFTSV enhance the propagation recombinant vesicular stomatitis virus vectors at assembly step. Viruses 15:800. doi:10.3390/v15030800 PMC10052781 36992507

[108] Westover JB, Jung KH, Alkan C, Boardman KM, Van Wettere AJ, Martens C, Rojas I, Hicks P, Thomas AJ, Saindane MT, Bluemling GR, Mao S, Kolykhalov AA, Natchus MG, Bates P, Painter GR, Ikegami T, Gowen BB. 2024. Modeling Heartland virus disease in mice and therapeutic intervention with 4’-fluorouridine. J Virol 98:e0013224. doi:10.1128/jvi.00132-24 PMC11019845 38511932

[109] Marqués M-C, Andreu-Moreno I, Sanjuán R, Elena SF, Geller R. 2024. An efficient plasmid-based system for the recovery of recombinant vesicular stomatitis virus encoding foreign glycoproteins. Sci Rep 14:14644. doi:10.1038/s41598-024-65384-8 PMC11199562 38918479

[110] Yun T, Park A, Hill TE, Pernet O, Beaty SM, Juelich TL, Smith JK, Zhang L, Wang YE, Vigant F, Gao J, Wu P, Lee B, Freiberg AN. 2015. Efficient reverse genetics reveals genetic determinants of budding and fusogenic differences between Nipah and Hendra viruses and enables real-time monitoring of viral spread in small animal models of henipavirus infection. J Virol 89:1242–1253. doi:10.1128/JVI.02583-14 PMC4300668 25392218

[111] Whitt MA. 2010. Generation of VSV pseudotypes using recombinant ΔG-VSV for studies on virus entry, identification of entry inhibitors, and immune responses to vaccines. J Virol Methods 169:365–374. doi:10.1016/j.jviromet.2010.08.006 PMC2956192 20709108

[112] Manzoni TB, Westover JB, Lundgreen KA, Hicks PD, Petch RJ, Ort JT, Weissman D, Fan SHY, Hensley SE, Pardi N, Gowen BB, Bates P. 2025. Homologous and heterologous vaccination regimens with mRNA and rVSV platforms induce potent immune responses against SFTSV glycoprotein. Viruses 17:1095. doi:10.3390/v17081095 PMC12390526 40872809

[113] Miao Y, Du Q, Zhang HG, Yuan Y, Zuo Y, Zheng H. 2023. Cycloheximide (CHX) chase assay to examine protein half-life. Bio Protoc 13:e4690. doi:10.21769/BioProtoc.4690 PMC10262206 37323633

[114] Schneider CA, Rasband WS, Eliceiri KW. 2012. NIH Image to ImageJ: 25 years of image analysis. Nat Methods 9:671–675. doi:10.1038/nmeth.2089 PMC5554542 22930834

[115] Manders EMM, Verbeek FJ, Aten JA. 1993. Measurement of co‐localization of objects in dual‐colour confocal images. J Microsc 169:375–382. doi:10.1111/j.1365-2818.1993.tb03313.x 33930978

[116] Livak KJ, Schmittgen TD. 2001. Analysis of relative gene expression data using real-time quantitative PCR and the 2(-Delta Delta C(T)) Method. Methods 25:402–408. doi:10.1006/meth.2001.1262 11846609

[117] Takada A, Robison C, Goto H, Sanchez A, Murti KG, Whitt MA, Kawaoka Y. 1997. A system for functional analysis of Ebola virus glycoprotein. Proc Natl Acad Sci USA 94:14764–14769. doi:10.1073/pnas.94.26.14764 PMC25111 9405687

[118] Lundu T, Tsuda Y, Ito R, Shimizu K, Kobayashi S, Yoshii K, Yoshimatsu K, Arikawa J, Kariwa H. 2018. Targeting of severe fever with thrombocytopenia syndrome virus structural proteins to the ERGIC (endoplasmic reticulum Golgi intermediate compartment) and Golgi complex. Biomed Res 39:27–38. doi:10.2220/biomedres.39.27 29467349

[119] Casey JR, Grinstein S, Orlowski J. 2010. Sensors and regulators of intracellular pH. Nat Rev Mol Cell Biol 11:50–61. doi:10.1038/nrm2820 19997129

[120] Cullen PJ, Steinberg F. 2018. To degrade or not to degrade: mechanisms and significance of endocytic recycling. Nat Rev Mol Cell Biol 19:679–696. doi:10.1038/s41580-018-0053-7 30194414

[121] Lokupathirage SMW, Tsuda Y, Ikegame K, Noda K, Muthusinghe DS, Kozawa F, Manzoor R, Shimizu K, Yoshimatsu K. 2021. Subcellular localization of nucleocapsid protein of SFTSV and its assembly into the ribonucleoprotein complex with L protein and viral RNA. Sci Rep 11:22977. doi:10.1038/s41598-021-01985-x PMC8626419 34836987

[122] Chen SY, Matsuoka Y, Compans RW. 1991. Golgi complex localization of the Punta Toro virus G2 protein requires its association with the G1 protein. Virology 183:351–365. doi:10.1016/0042-6822(91)90148-5 PMC7130718 1905078

[123] Sperber HS, Welke RW, Petazzi RA, Bergmann R, Schade M, Shai Y, Chiantia S, Herrmann A, Schwarzer R. 2019. Self-association and subcellular localization of Puumala hantavirus envelope proteins. Sci Rep 9:707. doi:10.1038/s41598-018-36879-y PMC6345964 30679542

[124] Cosson P, Letourneur F. 1994. Coatomer interaction with di-lysine endoplasmic reticulum retention motifs. Science 263:1629–1631. doi:10.1126/science.8128252 8128252

[125] Letourneur F, Gaynor EC, Hennecke S, Démollière C, Duden R, Emr SD, Riezman H, Cosson P. 1994. Coatomer is essential for retrieval of dilysine-tagged proteins to the endoplasmic reticulum. Cell 79:1199–1207. doi:10.1016/0092-8674(94)90011-6 8001155

[126] Hardt B, Bause E. 2002. Lysine can be replaced by histidine but not by arginine as the ER retrieval motif for type I membrane proteins. Biochem Biophys Res Commun 291:751–757. doi:10.1006/bbrc.2002.6515 11866428

[127] Dell’Angelica EC, Bonifacino JS. 2019. Coatopathies: genetic disorders of protein coats. Annu Rev Cell Dev Biol 35:131–168. doi:10.1146/annurev-cellbio-100818-125234 PMC7310445 31399000

[128] Stenbeck G, Harter C, Brecht A, Herrmann D, Lottspeich F, Orci L, Wieland FT. 1993. Beta’-COP, a novel subunit of coatomer. EMBO J 12:2841–2845. doi:10.1002/j.1460-2075.1993.tb05945.x PMC413534 8334999

[129] Cole NB, Sciaky N, Marotta A, Song J, Lippincott-Schwartz J. 1996. Golgi dispersal during microtubule disruption: regeneration of Golgi stacks at peripheral endoplasmic reticulum exit sites. MBoC 7:631–650. doi:10.1091/mbc.7.4.631 PMC275914 8730104

[130] Xu P, Hankins HM, MacDonald C, Erlinger SJ, Frazier MN, Diab NS, Piper RC, Jackson LP, MacGurn JA, Graham TR. 2017. COPI mediates recycling of an exocytic SNARE by recognition of a ubiquitin sorting signal. eLife 6. doi:10.7554/eLife.28342 PMC5663479 29058666

[131] Xie B, Guillem C, Date SS, Cohen CI, Jung C, Kendall AK, Best JT, Graham TR, Jackson LP. 2023. An interaction between β’-COP and the ArfGAP, Glo3, maintains post-Golgi cargo recycling. J Cell Biol 222:e202008061. doi:10.1083/jcb.202008061 PMC9960064 36811888

[132] van den Broek MF, Müller U, Huang S, Aguet M, Zinkernagel RM. 1995. Antiviral defense in mice lacking both alpha/beta and gamma interferon receptors. J Virol 69:4792–4796. doi:10.1128/jvi.69.8.4792-4796.1995 PMC189290 7609046

[133] Baldon LVR, de Mendonça SF, Ferreira FV, Rezende FO, Amadou SCG, Leite THJF, Rocha MN, Marques JT, Moreira LA, Ferreira AGA. 2022. AG129 mice as a comprehensive model for the experimental assessment of mosquito vector competence for arboviruses. Pathogens 11:879. doi:10.3390/pathogens11080879 PMC9412449 36015000

[134] Huber JP, David Farrar J. 2011. Regulation of effector and memory T-cell functions by type I interferon. Immunology 132:466–474. doi:10.1111/j.1365-2567.2011.03412.x PMC3075500 21320124

[135] McNab F, Mayer-Barber K, Sher A, Wack A, O’Garra A. 2015. Type I interferons in infectious disease. Nat Rev Immunol 15:87–103. doi:10.1038/nri3787 PMC7162685 25614319

[136] Bosco-Lauth AM, Calvert AE, Root JJ, Gidlewski T, Bird BH, Bowen RA, Muehlenbachs A, Zaki SR, Brault AC. 2016. Vertebrate host susceptibility to heartland virus. Emerg Infect Dis 22:2070–2077. doi:10.3201/eid2212.160472 PMC5189141 27869591

[137] Slough MM, Chandran K, Jangra RK. 2019. Two point mutations in old world hantavirus glycoproteins afford the generation of highly infectious recombinant vesicular stomatitis virus vectors. mBio 10. doi:10.1128/mBio.02372-18 PMC6325249 30622188

[138] Kumar R, Maji S, Tiwari S, Misra J, Gupta J, Kumar N, Gupta R, Jha NK. 2025. Precision vaccine design targeting the prefusion state of viral glycoproteins: advances in structural vaccinology. Biochem Pharmacol 242:117349. doi:10.1016/j.bcp.2025.117349 40972736

[139] Hsieh CL, Goldsmith JA, Schaub JM, DiVenere AM, Kuo HC, Javanmardi K, Le KC, Wrapp D, Lee AG, Liu Y, Chou CW, Byrne PO, Hjorth CK, Johnson NV, Ludes-Meyers J, Nguyen AW, Park J, Wang N, Amengor D, Lavinder JJ, Ippolito GC, Maynard JA, Finkelstein IJ, McLellan JS. 2020. Structure-based design of prefusion-stabilized SARS-CoV-2 spikes. Science 369:1501–1505. doi:10.1126/science.abd0826 PMC7402631 32703906

[140] Hsieh C-L, Rush SA, Palomo C, Chou C-W, Pickens W, Más V, McLellan JS. 2022. Structure-based design of prefusion-stabilized human metapneumovirus fusion proteins. Nat Commun 13:1299. doi:10.1038/s41467-022-28931-3 PMC8921277 35288548

[141] Ramos-Cela M, Forconi V, Antonelli R, Manenti A, Montomoli E. 2025. Exploring the use of viral vectors pseudotyped with viral glycoproteins as tools to study antibody-mediated neutralizing activity. Microorganisms 13:1785. doi:10.3390/microorganisms13081785 PMC12388780 40871289

[142] Jones SM, Ströher U, Fernando L, Qiu X, Alimonti J, Melito P, Bray M, Klenk H, Feldmann H. 2007. Assessment of a vesicular stomatitis virus–based vaccine by use of the mouse model of Ebola virus hemorrhagic fever. J Infect Dis 196:S404–S412. doi:10.1086/520591 17940977

[143] Westover JB, Rigas JD, Van Wettere AJ, Li R, Hickerson BT, Jung K-H, Miao J, Reynolds ES, Conrad BL, Nielson S, Furuta Y, Thangamani S, Wang Z, Gowen BB. 2017. Heartland virus infection in hamsters deficient in type I interferon signaling: protracted disease course ameliorated by favipiravir. Virology 511:175–183. doi:10.1016/j.virol.2017.08.004 PMC5623653 28865344

[144] Fujii H, Tani H, Egawa K, Taniguchi S, Yoshikawa T, Fukushi S, Yamada S, Harada S, Kurosu T, Shimojima M, Maeki T, Lim CK, Takayama-Ito M, Komeno T, Nakajima N, Furuta Y, Uda A, Morikawa S, Saijo M. 2022. Susceptibility of type I interferon receptor knock-out mice to heartland bandavirus (HRTV) infection and efficacy of favipiravir and ribavirin in the treatment of the mice infected with HRTV. Viruses 14:1668. doi: 10.3390/v14081668 10.3390/v14081668 PMC9416051 36016290

[145] Gunn BM, Yu WH, Karim MM, Brannan JM, Herbert AS, Wec AZ, Halfmann PJ, Fusco ML, Schendel SL, Gangavarapu K, et al.. 2018. A role for Fc function in therapeutic monoclonal antibody-mediated protection against Ebola virus. Cell Host & Microbe 24:221–233. doi:10.1016/j.chom.2018.07.009 PMC6298217 30092199

[146] Zhang A, Stacey HD, D’Agostino MR, Tugg Y, Marzok A, Miller MS. 2023. Beyond neutralization: Fc-dependent antibody effector functions in SARS-CoV-2 infection. Nat Rev Immunol 23:381–396. doi:10.1038/s41577-022-00813-1 PMC9761659 36536068

