# Supplementary material for: Mutations that positively affect Bandavirus glycoprotein function in VSV-vectored vaccines
Source: J Virol. 2026 Sep 24;100(10):e01311-26. doi: 10.1128/jvi.01311-26 (PMC13712768; doi:10.1128/jvi.01311-26)
Supplement: Supplementary file 1 [file jvi.01311-26-s0001.docx]

**Supplemental Figure 1. SFTSV glycoproteins with the K1071A+M749T mutation combination are more fusogenic.** Representative images of HeLa cells transfected with SFTSV WT, K1071A, or K1071A+M749T glycoproteins and subjected to acidic buffer treatment are shown in comparison to mock transfected cells.

**Supplemental Figure 2. Focus reduction neutralization tests specifically identify anti-HRTV neutralizing antibodies. (A)** Serum from mice vaccinated with rVSV-EBOV does not neutralize VSVΔG(HRTV) pseudotypes. **(B)** Serum from mice vaccinated with rVSV-HRTV mutants does not neutralize VSVΔG pseudotypes with the native VSV-G glycoprotein. Horizontal line indicates limit of detection (LOD) of the assay (20).
